# Spatial Proteomics Reveals Differences in the Cellular Architecture of Antibody-Producing CHO and Plasma Cell–Derived Cells

**DOI:** 10.1016/j.mcpro.2022.100278

**Published:** 2022-08-05

**Authors:** Robin Kretz, Larissa Walter, Nadja Raab, Nikolas Zeh, Ralph Gauges, Kerstin Otte, Simon Fischer, Dieter Stoll

**Affiliations:** 1 University of Applied Sciences Albstadt-Sigmaringen, Department of Life Sciences, Sigmaringen, Germany; 2 Department of Biology, Cell Biology, University of Constance, Konstanz, Germany; 3 NMI, Natural and Medical Sciences Institute at the University of Tuebingen, Reutlingen, Germany; 4 University of Applied Sciences Biberach, Institute of Applied Biotechnology, Biberach, Germany; 5 Boehringer Ingelheim Pharma GmbH & Co KG, Bioprocess Development Biologicals, Cell Line Development, Biberach, Germany

**Keywords:** subcellular proteomics, TMT, mass spectrometry, CHO cell line, plasma cell, AGC, automatic gain control, BH-FDR, Benjamini–Hochberg-corrected false discovery rate, CHO, Chinese hamster ovary, CID, collision-induced dissociation, DE, differentially expressed, DL, differentially localized, ECM, extracellular matrix, eIF4, eukaryotic translation initiation factor 4F, ER, endoplasmic reticulum, ERGIC, ER-Golgi intermediate compartment, GO, Gene Ontology, GOCC, Gene Ontology Cellular Component, HCD, higher energy collisional dissociation, IgG, immunoglobulin G, ISR, induced stress response, IQR, inter quartile range, KEGG, Kyoto Encyclopedia of Genes and Genomes, LFQ, label-free quantitation, LOPIT–DC, localization of organelle proteins by isotope tagging–differential centrifugation, MPC-11, Merwin plasma cell tumor-11, MS, mass spectrometry, mTOR, mammalian target of rapamycin, mTORC1, mammalian target of rapamycin complex 1, OST, oligosaccharly transferase, PBST, PBS with Tween-20, PCA, principal component analysis, PCD, plasma cell–derived, PM, plasma membrane, RTS, real-time search, SDC, sodium deoxycholate, SPS, synchronous precursor selection, SVM, support vector machine, TF, transcription factor, TMT, tandem mass tag, TRAPP, transport protein particle, UPR, unfolded protein response, UPSL, UniProt Subcellular location

## Abstract

Most of the recombinant biotherapeutics employed today to combat severe illnesses, for example, various types of cancer or autoimmune diseases, are produced by Chinese hamster ovary (CHO) cells. To meet the growing demand of these pharmaceuticals, CHO cells are under constant development in order to enhance their stability and productivity. The last decades saw a shift from empirical cell line optimization toward rational cell engineering using a growing number of large omics datasets to alter cell physiology on various levels. Especially proteomics workflows reached new levels in proteome coverage and data quality because of advances in high-resolution mass spectrometry instrumentation. One type of workflow concentrates on spatial proteomics by usage of subcellular fractionation of organelles with subsequent shotgun mass spectrometry proteomics and machine learning algorithms to determine the subcellular localization of large portions of the cellular proteome at a certain time point. Here, we present the first subcellular spatial proteome of a CHO-K1 cell line producing high titers of recombinant antibody in comparison to the spatial proteome of an antibody-producing plasma cell–derived myeloma cell line. Both cell lines show colocalization of immunoglobulin G chains with chaperones and proteins associated in protein glycosylation within the endoplasmic reticulum compartment. However, we report differences in the localization of proteins associated to vesicle-mediated transport, transcription, and translation, which may affect antibody production in both cell lines. Furthermore, pairing subcellular localization data with protein expression data revealed elevated protein masses for organelles in the secretory pathway in plasma cell–derived MPC-11 (Merwin plasma cell tumor-11) cells. Our study highlights the potential of subcellular spatial proteomics combined with protein expression as potent workflow to identify characteristics of highly efficient recombinant protein–expressing cell lines. Data are available *via* ProteomeXchange with identifier PXD029115.

## Highlights

• Subcellular proteome map of a CHO and a murine plasma cell–derived cell line.
• Detection of differentially localized proteins between antibody-secreting cells.
• Golgi apparatus and plasma membrane show differences in protein composition.
• Secretory pathway organelles show higher protein mass in MPC-11 cells.

## Graphical Abstract

**Figure:**
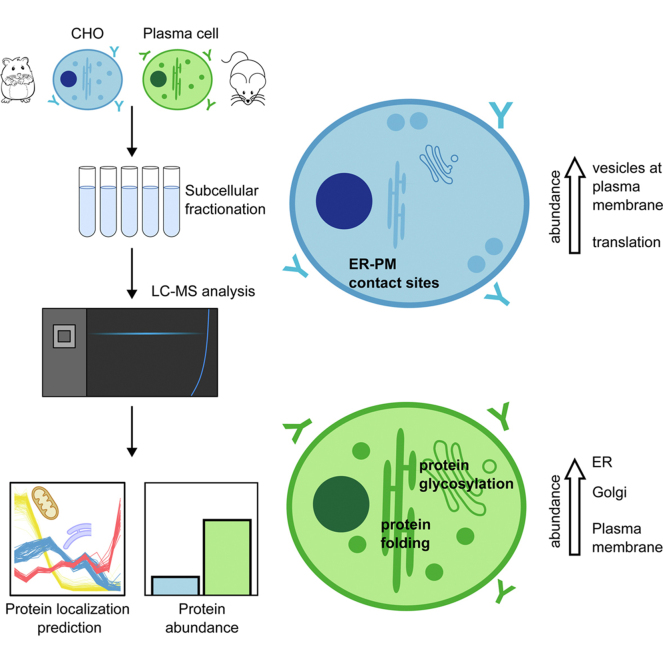

## In Brief

We used state-of-the-art protein spatial profiling together with MS-based protein expression analysis and subsequent bioinformatics analysis to elucidate the subcellular proteome organization and organelle architecture of two antibody-secreting cell lines, a CHO and a murine plasma derived cell line (MPC-11). Characterization of differentially expressed and localized proteins and their associated biological pathways allowed a systematic comparison of both cell lines and implicates further targets for CHO cell line engineering.

The usage of recombinant therapeutics, in particular, antibodies, has revolutionized modern medicine as their application has become essential in the treatment of severe diseases such as numerous cancer types and autoimmune diseases (1, 2). Today, these biopharmaceuticals are produced primarily in Chinese hamster ovary (CHO) cells (3), a cell line derived originally in 1957 (4). Since then, several variants of the original cell line were generated (*e.g.*, CHO-K1, CHO-S, CHO-DG44) providing the pharmaceutical industry and researchers with different expression systems to choose from. CHO cells show several properties favoring their application in biopharmaceutical production. They are able to grow to a high cell density in suspension cultures, show human-compatible glycosylation, and allow simplified downstream processing because of chemically defined serum-free media for cultivation. Furthermore, they show a safety viral profile, a good availability, and knowledge about specific culture media and supplements and enable usage of various expression systems for genetic engineering (*e.g.*, dihydrofolate reductase–deficient cell lines, glutamine synthetase–deficient cell lines) (3, 5, 6). Wide usage of CHO cells also brought extensive optimization effort to increase product yield by enhancing cell-specific productivity and/or by improving downstream processing of the product (7). Within the last years, optimization strategies have shifted from mostly media-driven optimization toward rational cell engineering since the advance of omics technologies enabled studies of whole-cell systems on various biological levels (8, 9, 10). CHO cellular pathways were studied using large-scale omics datasets (2, 11). Several studies showed that specific antibody productivity could be enhanced by applying the gained knowledge (12, 13, 14).

Rational cell engineering could gain further insights by comparing CHO cells with native antibody-producing cells, for example, plasma cells, stimulated B cells, or cell lines derived from plasma cell malignancies. Plasma cells acquire high secretion capacity upon their differentiation from naïve B-cells (15, 16). Numerous studies characterized the differentiation of naïve B-cells into plasma cells as highly complex mechanism initiated by a few master regulators, for example, interferon regulatory factor 4 (Irf4), B-lymphocyte-induced maturation protein 1 (Blimp1), and X-box binding protein 1 (Xbp1). Plasma cell differentiation reshapes and enlarges the endoplasmic reticulum (ER) and a large section of the downstream secretory pathway (16, 17, 18). Regulators of the unfolded protein response (UPR), such as Xbp1, activating transcription factor 4 (Atf4), and CCAAT/enhancer-binding protein homologous protein (Chop), already were targeted for overexpression in CHO cells with a maximum twofold increase in antibody production (14, 19, 20), indicating that cell engineering of CHO cells toward a plasma cell phenotype can enhance antibody secretion. However, it is suggested that additional pathways are needed for a plasma cell transformation, as for example, a recent study on murine B-cells indicates involvement of the mammalian target of rapamycin (mTOR) pathway in plasma cell differentiation (21, 22). In order to identify additional pathways and protein interactions relevant for establishing long-lasting high protein secretion, proteomic changes have to be elucidated not just on a total expression but also on spatial and temporal scales.

Whereas large proteomic studies already were conducted for CHO cell lines, the subcellular organization of the CHO cell proteome was not yet investigated until recently (23). Pathway signaling and protein function is critically affected by protein localization, since different subcellular compartments have distinct chemical milieus and provide proteins with different molecules to interact with. As it is suggested that between 10 and 50% of the proteins expressed in a cell at a given time point can be multilocal (24, 25), protein localization can be crucial for differences in cellular function (26). To study the proteome of subcellular structures, many different methods are available, such as immunofluorescence microscopy, proximity tagging, single-organelle purification, or protein correlation profiling (27, 28). The latter method uses high-throughput mass spectrometry (MS) to quantify protein abundance in several subcellular fractions, which usually are created by applying differential or density-based centrifugation to a cell lysate (24, 25, 29, 30). After quantification, machine learning methods and multivariate statistics are employed to correlate the fractionation distribution profiles of proteins of known subcellular localization to all proteins in the dataset. In this way, the subcellular distribution of a large portion of the cellular proteome can be inferred.

Here, we present to our knowledge the first high-resolution subcellular proteomic maps of an antibody-producing CHO and a plasma cell-derived (PCD) murine cell line (Merwin plasma cell tumor-11 [MPC-11]) using a modified version of the localization of organelle proteins by isotope tagging–differential centrifugation (LOPIT–DC) approach for map generation (24). Applying the same protocol to both cell lines enabled a full comparison of protein subcellular distributions across both cell lines. We found proteins associated with vesicle-mediated transport, transcription, and translation to be differentially localized (DL) within the cells. In addition, using the proteomic ruler approach (31) to combine protein expression and subcellular localization data, quantitative differences were revealed in organelle architecture, as MPC-11 cells showed elevated protein amounts for organelles of the secretory pathway (ER, Golgi apparatus, and plasma membrane [PM]).

Thus, our data represent a resource for analyzing antibody secretion in different cell lines from different species and complement orthogonal studies on CHO cells using state-of-the-art proteomics, metabolomics, and immunofluorescence methods for studying recombinant protein expression.

## Experimental Procedures

### Cell Culture

MPC-11 cells were bought from the European Collection of Authenticated Cell Cultures and cultured in Dulbecco's modified Eagle's medium (Biowest) media supplemented with 10% fetal bovine serum (Capricorn). Cells were cultivated statically at 37 °C, 5% CO_2_, and 85% humidity. CHO-K1 cells (Boehringer Ingelheim proprietary immunoglobulin G [IgG]-producing cell line) were grown in Boehringer Ingelheim proprietary media at 37 °C, 5% CO_2_, and 85% humidity under shaken conditions with 140 rpm (25 mm orbital shaker). Both cell lines were subcultured every 3 to 4 days and seeded with 5 × 10^5^ viable cells/ml. Viable cell concentration was determined by trypan blue exclusion (CEDEX XS; Roche Diagnostics).

### Protein A HPLC

To determine the antibody concentration, cell supernatant was filtered through 0.2 μm filter plates (AcroPrep Advance 96 Filter Plates; Pall Corporation) before assessing the antibody titer using the UltiMate 3000 System (Thermo Fisher Scientific) equipped with a Protein A POROS A 20 μm column (Thermo Fisher Scientific). The buffer for column equilibration and antibody binding consisted of 50 mM sodium hydrogen phosphate, 150 mM sodium chloride, titrated with sodium hydroxide to pH 7.5 and the elution buffer of 5.7% phosphoric acid, 150 mM sodium chloride with pH 2.5 (Carl Roth). Finally, protein concentration was detected by UV absorbance at 280 nm.

### Lysis and Lysate Fractionation

For the generation of subcellular proteome maps of the cell lines, a modified version of the LOPIT–DC protocol (24) was used, which combined the hypo-osmotic swelling and postlysis osmotic adjustment from the dynamic organellar maps (30) with the fractionation scheme from LOPIT–DC. Cells were cultured in triplicates until late exponential phase and then collected *via* centrifugation at 500*g* for 3 min. Cell pellets were washed three times with ice-cold PBS. Approximately 5 × 10^7^ cells were used for the generation of one replicate map. Cell pellets were resuspended in lysis buffer (50 mM sucrose, 0.5 mM magnesium chloride, 0.2 mM EGTA, 25 mM Tris, pH 7.4) and incubated on ice for 5 min to allow swelling of the cells. Cells were then lysed on ice by passing the suspension 15 times through a ball-bearing cell homogenizer (Isobiotec) with a 20 μm clearance size using a pair of 5 ml syringes. Afterwards, cOmplete protease inhibitor (Roche) was added to the lysate, and isotonic conditions were restored by adding hypertonic sucrose solution (2.5 M sucrose, 0.5 mM magnesium chloride, 0.2 mM EGTA, 25 mM Tris, pH 7.4). Lysates were cleared from debris and nonlysed cells by centrifugation at 300*g* for 5 min. This step was repeated two times. Lysate supernatants were fractionated into 10 fractions by a 9-step sequential differential centrifugation using an Optima MAX-XP Ultracentrifuge (Beckman). The centrifugation steps were as follows: 1000*g* for 10 min, 3000*g* for 10 min, 5000*g* for 10 min, 9000*g* for 15 min, 12,000*g* for 15 min, 15,000*g* for 15 min, 30,000*g* for 20 min, 79,000*g* for 43 min, and 120,000*g* for 45 min. After each centrifugation step, supernatants were collected for the following centrifugation step and pellets were resuspended in 200 μl membrane solubilization buffer (50 mM Hepes, pH 8.5, 0.2% SDS, 8 M urea) + cOmplete protease inhibitor. The final supernatant was collected as 10th fraction. Each fraction then was sonicated for 3 min on ice.

Unfractionated cell lysates were generated by collecting 10^6^ cells in late exponential phase *via* centrifugation at 500*g* for 3 min. Cells were washed as described previously and resuspended in 400 μl sodium deoxycholate (SDC) buffer (4% SDC [w/v], 100 mM Hepes; pH 8.5) followed by 5 min incubation on ice. Afterward, cells were heated to 95 °C for 5 min and 550 rpm, allowed to cool down and sonicated for 5 min on ice. 250U Benzonase (Merck KGaA) was added to degrade DNA and reduce viscosity.

### LC–MS Sample Preparation and Tandem Mass Tag Labeling

Protein concentration of fractions and whole-cell lysates were measured by Pierce bicinchoninic acid assay (Pierce, Thermo Fisher Scientific). For LC–MS sample preparation, 50 μg protein per fraction or whole-cell lysate was used. Samples were reduced by addition of DTT to a final concentration of 10 mM and incubated for 2 h at room temperature. Samples were alkylated with 25 mM iodoacetamide for 2 h at room temperature in the dark. Afterward, samples were diluted 1:1 with LC–MS grade water and proteins were precipitated by adding six volumes of ice-cold acetone for overnight incubation at −20 °C. Samples were centrifuged at 10,000*g* at 4 °C for 5 min, and the supernatants were discarded. Protein pellets were air-dried for 5 min and then resuspended in 50 mM Hepes (pH 8.5). rLysC (Promega) was added at an enzyme to protein ratio of 1:50 for a first proteolysis step at 37 °C for 3 h. Then, trypsin (Thermo Fisher Scientific) was added at the same enzyme to protein ratio as rLysC for overnight proteolysis at 37 °C. Digests of whole-cell lysates were acidified by addition of formic acid (end concentration 0.1% [v/v] and directly subjected to LC–MS analysis [see later]).

Peptide fractions of one spatial map then were labeled using tandem mass tag (TMT) TMT10plex reagent (Thermo Fisher Scientific) according to the manufacturer’s instruction. Each of the 10 fractions was labeled with one TMT-plex, respectively. In order to avoid channel-specific bias, TMT labels and fractions were randomized prior to labeling (see supplemental Table S3 for labeling scheme). Briefly, TMT reagents were equilibrated to room temperature prior to labeling and resuspended in 41 μl of anhydrous acetonitrile (MeCN). Peptide fractions were then combined with TMT reagents and incubated for 1 h at room temperature. Afterward, the reaction was quenched by adding 8 μl of 5% hydroxylamine followed by additional 15 min incubation time at room temperature. Subsequently, TMT-labeled fractions were combined into one multiplex per replicate.

The multiplexed samples were dried by vacuum centrifugation and cleaned using AttractSPE Spin-C18.T1.96 cartridges (AFFINISEP). For this, labeled samples were resuspended in 50 mM Hepes (pH 8.5) diluted 4:1 with 2% trifluoroacetic acid (v/v) + 20% MeCN (v/v) and applied to the AttractSPE cartridges. Peptides were eluted using 70% MeCN (v/v) + 0.1% formic acid (v/v), combined into one Eppendorf tube as one TMTplex and again dried by vacuum centrifugation.

### Offline High-pH Fractionation

TMT-multiplexed samples were fractionated by high-pH reverse-phase chromatography. The TMT-labeled samples were resuspended in 20 mM ammonium formate pH 10 (solvent A) and completely injected onto a XBridge BEH XP C18 (150 mm × 2.1 mm i.d., 2.5 μm particle size) column (Waters) installed on a 1260 Infinity II LC-System (Agilent) equipped with a diode array detector. Peptides were separated using a binary buffer system consisting of solvent A (20 mM ammonium formate pH 10 [v/v]) and solvent B (80% MeCN [v/v] + 20 mM ammonium formate pH 10 [v/v]) and a linear gradient from 5% to 75% solvent B in 50 min followed by a wash step with 99% solvent B for 10 min. The flow rate was 0.25 ml/min. Fractions were collected every minute, freeze dried, and combined into 19 or 20 fractions for downstream MS analysis as follows. Fractions 1 to 15 were combined into one fraction, fractions 16 to 49 were combined orthogonally (16 + 33, 17 + 34, etc.) into 17 fractions, and the remaining fractions 50 to 70 were again combined into one single fraction.

### Chromatography and MS

Combined TMT-peptide fractions from offline high-pH fractionation were resuspended in 0.1% formic acid (v/v) and subsequently analyzed by LC–MS using an Orbitrap Eclipse Tribrid Instrument coupled to an UltiMate 3000 RSLCnano system (Thermo Fisher Scientific).

Approximately 1 μg of a fraction was injected into the HPLC system and trapped on an Acclaim PepMap C18 5 mm × 0.3 mm i.d., 5 μm particle size, 100 Å pore size trapping column using 2% MeCN (v/v) + 0.05% trifluoroacetic acid as solvent at 50 μl/min flow rate for 5 min. Afterward, the injection valve was switched to the analytical column. Peptides were eluted from the trapping column and separated using an Acclaim PepMap RSLC C18 column, 50 cm × 75 μm i.d., 2 μm particle size, 100 Å pore size (Thermo Fisher Scientific). Nano LC-gradient parameters were as follows: solvent A: 0.1% formic acid (v/v), solvent B: 80% MeCN (v/v) + 0.1% formic acid (v/v). A linear gradient from 2 to 40% solvent B in 95 min was used at a flow rate of 0.3 μl/min followed by a wash step for 10 min at 99% solvent B. Afterward, an equilibration step of 10 min at 2% solvent B was included. Column oven temperature was 55 °C. Total run time was 120 min.

The mass spectrometer was operated in positive data-dependent acquisition mode. Peptides were measured using synchronous precursor selection–MS^3^ (SPS–MS^3^) acquisition mode. For SPS–MS^3^ data acquisition, MS^2^ acquired data were searched against an organism-specific proteome database using the real-time search (RTS) method implemented in the Xcalibur control software of the mass spectrometer. For MPC-11 cells, the mouse SwissProt proteome database (32) (proteome ID: UP000000589, downloaded from www.uniprot.org, June 13, 2021) and for CHO-K1 cells the CHO-K1 UniProt Knowledgebase database (proteome ID: UP000001075, downloaded from www.uniprot.org, June 14, 2021) with human immunoglobulin heavy and light chain sequences added were used. The MS parameters were as follows:

MS1: *detector type*: Orbitrap; *resolution*: 120,000; *scan range* (*m/z*): 380 to 1600; *RF lens* (%): 30; *automatic gain control (AGC) target*: standard; *maximum injection time mode*: auto; *microscans*: 1; and *data type*: profile. *Monoisotopic peak determination*: peptide; *include charge state(s)*: 2 to 6; *exclude after n times*: 1; *exclusion duration*: 70 s; *mass tolerance*: ppm, *low*: 10, *high*: 10; *exclude isotopes*: true; *perform dependent scan on single-charge state per precursor only*: true; *intensity threshold*: 5.0e3; *data-dependent mode*: cycle time; and *time between master scans* (seconds): 2.5.

MS2: *isolation mode*: quadrupole; *isolation window* (*m/z*): 0.7; *isolation offset*: off; *activation type*: collision-induced dissociation (CID); *CID energy mode*: fixed; *CID collision energy* (%): 35; *CID activation time* (milliseconds): 10; *activation Q*: 0.25; *detector type*: ion trap; *ion trap scan rate*: turbo; *scan range mode*: auto; *AGC target*: standard; *maximum injection time*: auto; *microscans*: 1; *data type*: centroid; *mass range* (*m/z*): 400 to 1200; *reagent*: TMT; *exclusion mass width*: *m/z*, *low*: 50, *high*: 5.

RTS: *enzyme*: trypsin; *static modifications*: carbamidomethyl (C) (mass 57.0215), TMT6plex (Kn) (mass 229.1629); *variable modifications*: oxidation (M) (mass 15.9949); *maximum missed cleavages*: 1; *maximum variable mods/peptide*: 2; *SPS mode*: true; *maximum search time* (ms): 35; *Xcorr*: 0; *dCn*: 0; and *precursor PPM*: 10; *charge state*: 1.

MS3: *MS*^*n*^ *level*: 3; *SPS*: true; *number of SPS precursors*: 10; *MS isolation window* (*m/z*): 0.7; *MS2 isolation window* (*m/z*): 3; *isolation offset*: off; *activation type*: higher energy collisional dissociation [HCD]; *HCD collision energy* (%): 65; *detector type*: Orbitrap; *resolution*: 30,000; *TurboTMT*: TMT and TMTpro reagents; *scan range mode:* define *m/z* range; *scan range* (*m/z*): 100 to 500; *AGC target*: custom; *normalized AGC target* (%): 500; *maximum injection time*: auto; *microscans*: 1; *data type*: centroid; *number of dependent scans*: 10.

Approximately 1 μg peptides of whole-cell lysate samples were injected into the HPLC system and separated using the same analytical column as described previously. The gradient increased from 2 to 5% solvent B in 5 min, from 5 to 35% solvent B in 190 min, and from 35 to 60% solvent B in 25 min. A wash step at 99% solvent B and a subsequent equilibration step at 2% solvent B was included. The flow rate was 0.3 μl/min, and the total runtime was 245 min. The mass spectrometer was operated in positive data-dependent acquisition mode, and the top 15 precursor peptides were selected for MS–MS measurement with HCD at 30% normalized collision energy. Precursor ions were measured with a resolution of 120,000 over a scan range of 350 to 2000 *m/z*. AGC target was set to “Standard” and injection time to “Auto.” Ions with a charge state of 2 to 4 and minimum intensity of 2.5e4 were selected for fragmentation. Fragment ions were isolated with an isolation window of 1.4 *m/z* and measured at an Orbitrap resolution of 15,000.

### Experimental Design and Statistical Rationale

Each cell line was measured in three independent biological replicates. For each replicate, cell culture, lysis, fractionation by ultracentrifugation, high-pH fractionation, LC–MS analysis, and MS data processing were conducted independently from other replicates on different days. Single replicate maps were used to assess robustness of the workflow, spatial resolution, and machine learning performance as described in more detail in the respective experimental procedures and result sections. As described (30), single replicate maps were merged into one combined map for each cell line in order to improve spatial resolution and machine learning performance. For the detection of DL proteins between cell line maps, replicate maps were used to calculate statistical significance of changes in Mahalanobis distances for each protein (two-sided Student’s *t* test, Benjamini–Hochberg-corrected false discovery rate [BH-FDR] = 0.05). Differentially expressed (DE) proteins were determined by applying DEqMS (33) (BH-FDR = 0.05) to independent biological triplicates of whole-cell lysates.

### MS Data Processing

MS raw data were processed using MaxQuant, version 1.6.17.0 (Max Planck Institute of Biochemistry, Martinsried) (34) and its built-in search engine Andromeda (35). MS data were searched against the same databases used for RTS in MS data acquisition (see aforementioned) with added reverse decoy sequences (*Mus musculus*: 50,736 sequences; proteome ID: UP000000589, downloaded from www.uniprot.org, June 13, 2021; CHO-K1: 47,778 sequences, proteome ID: UP000001075, downloaded from www.uniprot.org, June 14, 2021). Sample type was set to “reporter ion MS3,” and all TMT10plex labels for both lysine residues and N termini were selected. Batch-specific correction factors were used to correct the reporter ion intensities. Matching between runs was not activated. Trypsin/P was selected as enzyme for proteolysis with a maximum of two missed cleavage sites allowed. As variable modifications, oxidation of methionine residues and acetylation of N termini were selected. Carbamidomethylation of cysteine residues was specified as fixed modification. A maximum of five modifications per peptide was allowed. Precursor ion search tolerance was set to 4.5 ppm, whereas fragment ion search tolerance was set to 0.5 Da. Both peptide and protein identifications were filtered by applying a 0.01 FDR cutoff as determined by target-decoy search against a database containing the reversed protein sequences. All other parameters were kept at the default value. The “proteinGroups.txt” output file from MaxQuant was used for further data processing. Replicate sample sets were analyzed separately.

For whole-cell lysates, sample type was set to “Standard” and label-free quantitation (“LFQ”) as well as “Match between runs” was enabled. Mass spectra were searched against an artificially constructed database containing sequence identical peptides of 23,563 proteins between *Cricetulus griseus* and *M. musculus* with a length of 5 to 25 amino acids (36) and their reversed decoy sequences (number of searched sequences: 47,126). Replicate samples were processed together. All other parameters were kept at their default values.

Initial data processing of MaxQuant “proteinGroups.txt” files was performed within Perseus software, version 1.6.14.0 (Max Planck Institute of Biochemistry, Martinsried) (37). First, proteins that were identified as “only identified by site,” “potential contaminant,” or by matching to the reverse decoy database were removed from the dataset. Second, only proteins with complete TMT reporter ion series were retained. For combined maps, only proteins with complete TMT reporter ion series over all replicates of one cell line were retained (30 TMT ion channels). Protein profiles then were calculated as ratio of the summarized intensity over all TMT intensities for the respective protein. Combined maps were generated by calculating the mean protein profiles over the three replicates. Processing of whole-cell lysates “proteinGroups.txt” output files was conducted accordingly to TMT data. Proteins were retained, if they were found in all three replicates of the respective cell line and LFQ intensity values were log2 transformed. Missing values were imputed by sampling from a 1.8× SD left-shifted normal distribution with 0.3 times the original SD.

### Generation of Spatial Maps by Machine Learning

Spatial proteome maps were generated using the R statistical language, version 3.6.3 (38), Bioconductor (39) packages, MSnbase, version 2.15.7 (40) and pRoloc, version 1.30.0 (41) as described (24). Processed data were loaded into R and annotated with an organelle-specific protein marker set, which was generated for each cell line, respectively. The base for both marker sets was the murine-specific organelle protein marker set incorporated within the pRoloc package (accessible *via* pRolocmarkers() function). Additional proteins were added based on the literature and the organelle marker set proposed by Borner *et al.* (42). The CHO-specific organelle protein marker set was generated by searching the murine marker set against the UniProt knowledge base *C. griseus* CHO-K1 database using BLAST+ (43). Protein hits with sequence identity of ≥80% were retained, and the hit with lowest e-value was chosen as homologous protein. Finally, the murine marker set was filtered to only contain proteins for which a CHO homologous protein was found. Both marker sets contained 719 organelle marker proteins spread across 11 different subcellular localizations (cytosol, ER, Golgi apparatus, lysosome, mitochondrion, nucleus—chromatin, nucleus—nonchromatin, peroxisome, PM, proteasome, and ribosome) (supplemental Table S2).

The separation and resolution of subcellular clusters generated by organelle marker proteins were assessed by quantifying the euclidean intercluster distance normalized to the intracluster distance of the respective clusters using the QSep module within the pRoloc package in R.

Machine learning algorithms were implemented using the e1071 package, version 1.7.6 in R (44) as interface to libsvm, version 2.6 (45). Organelle marker sets were used to train a support vector machine (SVM) classifier with radial basis function kernel in order to predict subcellular locations across the whole proteome map. Each dataset was split up into a training set (80%) and a testing set (20%), and the cost and sigma hyperparameters of the SVM classifier were optimized *via* grid search with 100 times internal crossvalidation. Grid search was conducted from values 20 to 60 in increments of two for cost and from values 0.01 to 100 in multiples of 10 for sigma. To avoid overfitting, fivefold crossvalidation was performed on the whole dataset. Macro F1 scores (harmonic mean of precision and recall) were calculated to determine the best hyperparameter set for each map, respectively. For all CHO-K1 single-replicate data, optimal sigma was 1 and cost 54. For MPC-11 single-replicate data, sigma varied between 0.1 and 1 and cost between 26 and 60, respectively. A sigma of 1 and cost of 56 was determined as optimal hyperparameter set for MPC-11 and a sigma of 1 and cost of 48 for CHO-K1 cells, respectively. Then, SVM-based classification was applied to the entire map. Classification threshold was set to SVM score ≥0.7 for each cluster.

### Concordance Analysis

Pairwise map concordance analysis was performed for every single replicate map pair. Prior to concordance analysis, marker proteins were removed from each dataset. Concordance of two maps was calculated as the fraction of proteins with identical classification in two maps in respective to the total overlap of proteins between the two maps. Map concordance further was stratified in relation to SVM scores. For this, we defined four prediction accuracy classes: very high (SVM score ≥0.9), high (SVM score 0.7–0.9), medium (SVM score 0.5–0.7), and low (SVM score <0.5). Pairwise map concordance was calculated for each map pair and prediction class.

### Classification Comparison to Published Datasets and Databases

Combined map classifications were compared with two pre-existing datasets from murine primary neurons (46) and murine pluripotent stem cells (29). For comparison to murine datasets, the CHO-K1 dataset was annotated with UniProt accessions from homologous murine proteins as determined by BLAST+ search (sequence identity ≥0.8, lowest e value). Comparison to the pluripotent stem cell dataset directly was carried out by calculating the fraction of cluster pairs over the total overlap of both datasets. Prior to comparison, the primary neurons dataset was filtered for proteins in the classification prediction classes “high” and “medium,” as they are roughly comparable to our classification threshold of SVM score ≥0.7. Comparison was then carried out analogous to the dataset from pluripotent stem cells.

Combined maps further were compared with their Gene Ontology (GO) Cellular Component (GOCC) (47, 48) and UniProt Subcellular Location annotations. Datasets were annotated with GOCC and UniProt Subcellular Location data using Perseus software. Only proteins with an annotation for one certain subcellular location were counted for the calculation of the proportion of proteins with distinct annotation terms to the total cluster size.

### Detection of DL Proteins

Proteins with different subcellular distributions between CHO-K1 and MPC-11 subcellular maps were detected by a two-stage approach. First, we qualitatively examined the combined maps of both cell lines and determined proteins classified to different compartments as being differentially located. To compare the nuclear clusters between cell lines, we combined the nuclear compartments of CHO-K1 maps to yield one “Nucleus” compartment. Second, for all other proteins, we used an approach similar to magnetic resonance analysis as described (30, 46). For each protein, we calculated the Mahalanobis distance to the mean of the quantitative profiles of each SVM-classified subcellular compartment. For the calculation of the Mahalanobis distances, a robust estimate for the covariance matrix was used by implementing minimum covariance determinant. Thus, the localization of each protein was described as a 10-point profile of distances to each compartment in its respective map. In addition, we determined the closest compartment of each protein in each map as the compartment with smallest Mahalanobis distance. Each distance was tested on statistical significance between CHO-K1 and MPC-11 maps (Student’s *t* test, BH-FDR = 0.05). Finally, we determined a protein to differ in localization if the closest compartment of a protein changed between maps and if both distances to these closest compartments were significantly different between cell lines.

### GO Enrichment Analysis

Proteins were annotated with GO and Kyoto Encyclopedia of Genes and Genomes terms and subjected to enrichment analysis *via* Fisher’s exact test (BH-FDR = 0.02) within Perseus software. Respective protein lists with UniProt accession numbers were tested against the *M. musculus* SwissProt database as background.

### Copy Number Estimation by Proteomic Ruler

The proteomic ruler approach (31) implemented in Perseus software was used to derive protein copy numbers per cell and concentration estimates from whole-cell lysates and TMT-labeled fractions. Preprocessing of whole-cell lysates and TMT-labeled fractions was conducted according to Ref. (46). Briefly, for whole-cell lysates, log2 LFQ intensity values were used. For TMT-labeled fractions, TMT channels per replicate map were normalized to the total channel intensity and subsequently weighted by fraction protein yield. To obtain a single value per protein, weighted fraction intensities were summed up. For protein copy number estimation, ploidy of MPC-11 cells was set to three according to their hyperdiploid karyotype (49). Ploidy of CHO-K1 was set to 1.76 (ratio of CHO-K1 genome size and *M. musculus* genome size 2.399 Gbp, RefSeq Assembly GCF_000223135.1, release July 12, 2020, 2.72 Gbp, RefSeq Assembly GCF_000001635.27, release July 24, 2020 multiplied by 2). Replicates were processed separately. Remaining values were kept at their default setting. For each protein, copy numbers were multiplied by the protein’s molecular weight to obtain the protein mass/cell.

In order to obtain organelle protein masses, normalized and weighted TMT-intensity values from fractions were combined into a “nuclear,” “organelle,” and “cytosol” fractions as follows: “nucleus”: fraction 1, “organelle”: sum of fraction 2 to 9, and “cytosol”: fraction 10. Values from these fractions were added and expressed as ratio of the sum. These relative values were used in order to weight protein masses according to the assigned organelle (see supplemental Table S1 for a detailed explanation). Organelle protein masses then were calculated by summation of weighted protein masses/cell and expressed as ratio to the total protein mass.

### DE Proteins

DE proteins were determined using the Limma, version 3.42.2 (50) and DEqMS, version 1.4.0 (33) packages within R. Variance of samples was estimated using the “razor + unique peptide” columns from the “proteinGroups.txt” output table of MaxQuant. *p* Values were corrected for multiple sample testing (BH-FDR = 0.05).

### DigiWest Analysis

Fractions obtained by sequential centrifugation were analyzed using a multiplexed bead-based version of the Western blot (DigiWest) (51). Briefly, fractions were dried in a vacuum concentrator (Bachofer), resuspended in 2× lithium dodecyl sulfate, and subjected to SDS-PAGE with subsequent Western blotting *via* the NuPAGE system (Thermo Fisher Scientific). Membrane-bound proteins were washed with PBST (0.1% Tween-20, PBS) prior to biotinylation with 50 μM NHS-PEG12-Biotin (Thermo Fisher Scientific) in PBST for 1 h. Membranes were washed again with PBST and dried over night. Afterward, each Western blot lane was cut into 96 stripes (width = 0.5 mm), and the stripes were distributed into the wells of a 96-well plate (Greiner Bio-One). Elution buffer (8 M urea, 1% Triton X-100 in 100 mM Tris–HCl, pH 9.5) was used to elute proteins from the stripes. Eluates were diluted 1:10 with 5% bovine serum albumin in PBST, 0.02% sodium azide before adding NeutrAvidin-coated MagPlex beads (Luminex) of a distinct color ID to each well for overnight incubation. Remaining NeutrAvidin-binding sites were blocked with 500 μM PEG12-biotin in PBST. All bead-bound fractions of one lane were collected and pooled to reconstruct the original Western blot lanes.

For protein detection, 5 μl of the pooled beads were diluted with 50 μl assay buffer (blocking reagent for ELISA [Roche, Switzerland] supplemented with 0.2% milk powder, 0.05% Tween-20, and 0.02% sodium azide) and transferred into a 96-well plate. After discarding the buffer, 30 μl of primary antibody diluted in assay buffer were added per well and the plate was then incubated at 15 °C overnight. Wells were washed twice with PBST before 30 μl of phycoerythrine-labeled species–specific IgG secondary antibody diluted in assay buffer was added to the wells for 1 h at 23 °C. Afterward, wells again were washed twice with PBST. Plate readouts were conducted on a Luminex FlexMAP 3D (Thermo Fisher Scientific). Data were processed using an Excel macro-based algorithm for peak identification at the provided molecular weight of each antibody, respectively. Protein signal intensities were calculated as integrals of peak areas after local background subtraction. Resulting signals were normalized to the total protein amount loaded onto the beads. Quantitative protein signals were translated into black and white color values to represent the visual depiction of a usual Western blot. A list of all primary and secondary antibodies and their dilutions used is provided in supplemental Table S8.

## Results

### Generation of Spatial Proteome Maps for CHO-K1 and MPC-11 Cells by LOPIT–DC

**Fig. 1:**
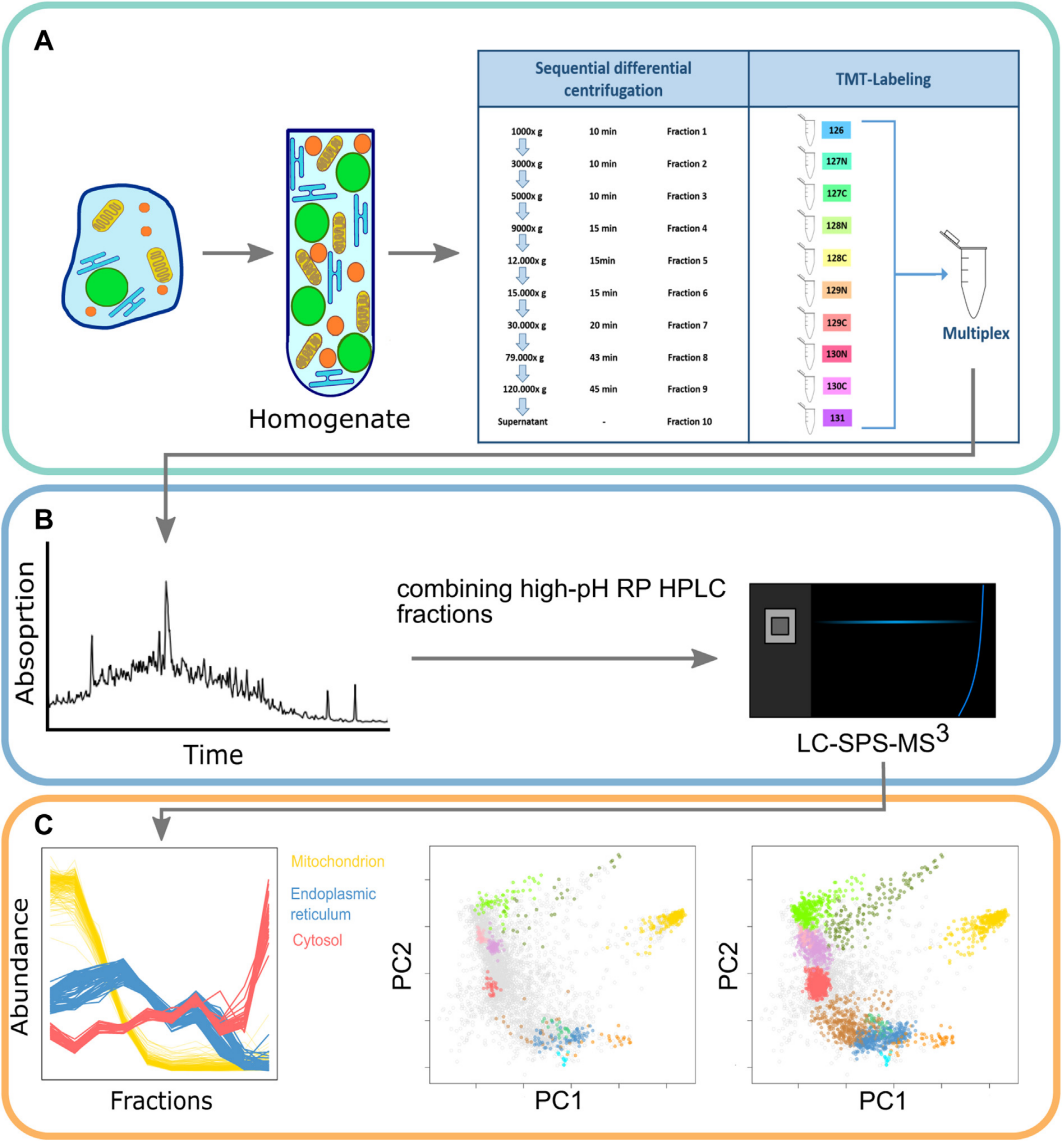
**LOPIT–DC workflow overview.***A*, cultivated cells were harvested and homogenized by gentle mechanical lysis using a ball-bearing cell homogenizer. Homogenates with still intact organelles were cleared up initially from cell debris by centrifugation at low speed before they were subjected to a 10-step differential centrifugation scheme. The 10-step differential centrifugation enabled crude fractionation of organelles, which were recovered by collecting the pellet after each step. After the last centrifugation step, the supernatant was collected as the 10th fraction. Fractions were reduced, alkylated, digested, and peptides were labeled with tandem mass tags (TMT 10-plex). *B*, combined TMT-labeled peptides were fractionated *via* high-pH reverse-phase HPLC and afterward combined orthogonally into 20 fractions, which in turn were subjected to liquid chromatography–synchronous precursor selection–mass spectrometry (LC–SPS–MS^3^) with real-time search. *C*, TMT reporter ions were quantified over 10 organelle fractions (*left*), and profiles of organelle marker proteins were visualized by principal component analysis (PCA, *middle*) revealing subcellular compartments within the proteomic dataset. Support vector machine (SVM) algorithms then were trained on organelle marker proteins to classify over 50% of proteins of the dataset to one of the subcellular compartments. LOPIT–DC, localization of organelle proteins by isotope tagging–differential centrifugation.

In order to analyze the subcellular proteome, a modified version of the LOPIT–DC protocol (24) was applied in independent triplicates to CHO-K1 and MPC-11 cells, respectively (Fig. 1). Subcellular fractionation was highly reproducible for both cell lines and resulted in protein yields above 50 μg per fraction (supplemental Fig. S1). Subsequent LC–SPS–MS^3^ analysis with integrated RTS identified and quantified over 6000 unique proteins in total for each respective cell line (6185 proteins for MPC-11 and 6779 proteins for CHO-K1 cells). Proteins with a full reporter ion series over all replicates of a cell line were merged into one dataset, yielding a combined dataset of 4102 proteins for MPC-11 and 5027 proteins for CHO-K1 cells, respectively.

**Fig. 2:**
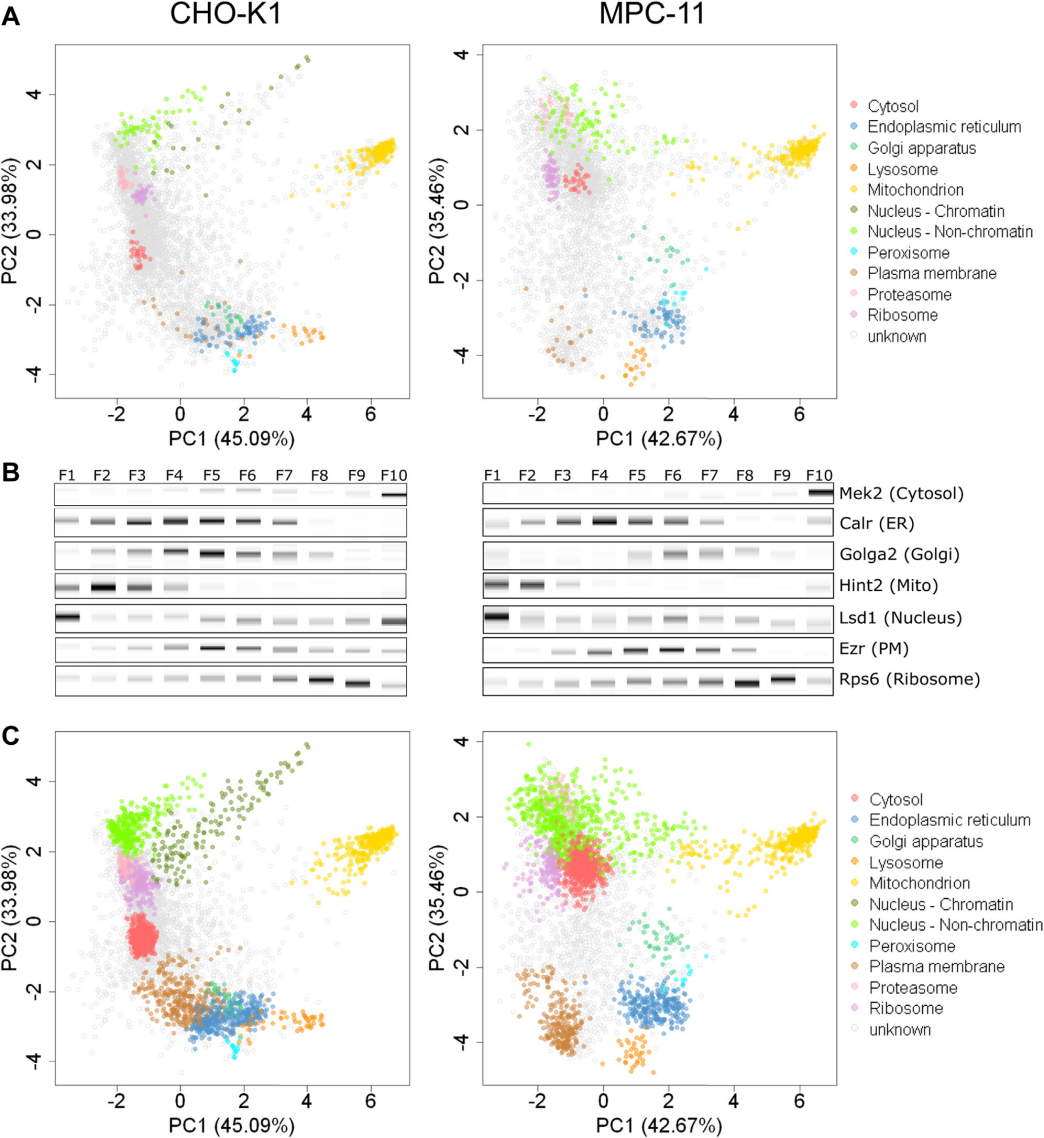
**Subcellular proteomics datasets for CHO-K1 and MPC-11 cells.***A*, principal component analysis (PCA) of combined datasets for CHO-K1 (*left*) und MPC-11 cells (*right*). Organelle marker proteins for 10 (MPC-11, no nucleus—chromatin cluster) respective 11 subcellular compartments (CHO-K1) were colorized. *B*, multiplexed DigiWest Western blot diagrams of selected organelle marker proteins across 10 centrifugal fractions of CHO-K1 (*left*) and MPC-11 (*right*) cells. Digital values were converted into *black* and *white colors* to reconstruct Western blot bands. Each organelle marker protein showed a distinct fractionation pattern across all fractions. *C*, subcellular proteomics datasets for CHO-K1 (*left*) and MPC-11 (*right*) cells after classification by support vector machine (SVM) algorithms. The SVM algorithm was trained on organelle marker proteins and optimized algorithm used for classification of all quantified proteins. Classification threshold was set to an SVM score ≥0.7. Proteins are colorized according to their subcellular localization prediction for both cell lines, respectively. CHO, Chinese hamster ovary; ER, endoplasmic reticulum; Mito, mitochondrion; MPC-11, Merwin plasma cell tumor-11; PM, plasma membrane.

Principal component analysis plots revealed distinct clustering of organelle marker proteins (supplemental Table S2) for 10 subcellular compartments (cytosol, ER, Golgi apparatus, lysosome, mitochondrion, nucleus, peroxisome, PM, proteasome, and ribosome) in MPC-11 and CHO-K1 cells (Fig. 2*A*). An additional nucleus–chromatin cluster revealed subnuclear resolution in CHO-K1 cells. Fractionation patterns of organelle marker proteins were verified by multiplexed DigiWest Western blot analysis (51) (Fig. 2*B*). Subcellular resolution of single replicate and combined maps was quantified by calculating normalized intercluster distances in euclidean space using the QSep module within the pRoloc package (41) in R (38). Intercluster distances showed high subcellular resolution for all 10 respective 11 subcellular compartments, with combined datasets from the three independent analyses showing highest overall mean cluster distances (supplemental Fig. S2).

In order to predict the subcellular localization for a large proportion of proteins, organelle marker proteins were used to train an SVM algorithm. Mean macro F1 scores were calculated after parameter optimization and were at 0.95 for both combined maps, indicating that nearly all organelle marker proteins were consistently assigned to the correct organelle cluster during crossvalidation (a maximum mean macro F1 score of 1 means all marker proteins are assigned to the correct cluster in all iterations). Similar to subcellular resolution, the combination of single replicates also increased classifier performance (supplemental Fig. S3*A*). Quantifying concordance in respective to stratified SVM score classes revealed high correlation of classification reproducibility and SVM scores (supplemental Fig. S3*B*) with the two prediction classes “very high” and “high” displaying mean concordances of >99% (“very high”) and >90% (“high”). Simultaneously, lower concordance in the classes “medium” and “low” indicated increasing uncertainty of protein classification with decreasing SVM score. Thus, we used an SVM score of ≥0.7 as cutoff for protein classification in all single and combined spatial maps.

With these stringend cutoff criteria, a total of 2444 (49%) out of 5027 proteins were assigned to 11 different subcellular compartments in CHO-K1, and 2577 (63%) out of 4102 proteins could be assigned to 10 different subcellular compartments in MPC-11 cells (Fig. 2*C*, supplemental Tables S3 and S4). We assessed our prediction accuracy by comparing protein classification to two published profiling spatial proteomics datasets of murine cells (29, 46) as well as to protein localization annotations in the UniProt (32) and GO database (GOCC) (47, 48). Comparison showed high concordance of both CHO-K1 and MPC-11 datasets to database entries (supplemental Fig. S4) as well as to the dataset reported by Christoforou *et al.* (29) (supplemental Fig. S5*A*). In addition, datasets showed high accordance to classifications reported by Itzhak *et al.* (46) for proteins classified to the ER, Golgi apparatus, mitochondrion, and peroxisome (supplemental Fig. S4*B*). Itzhak *et al.* (46) combined most of cytosolic, nuclear, and ribosomal proteins within a “large protein complexes” cluster, containing 42% of classified proteins within the dataset. Most of the proteins we predicted to be localized to the cytosol, nucleus, or ribosomes showed ”large protein complex” classification within the Itzhak *et al.* (46) dataset, depicting good agreement of the protein classifications. Taken together, these results indicate a high accuracy of predicted subcellular localization for a minimum of 50% of the here reported proteomes for CHO-K1 and MPC-11 cells.

However, we note that such classifications, as reported here, do not represent the full diversity of subcellular compartments. Therefore, we are aware that our data show changes in protein localization but that there is always the possibility of misclassification of proteins because of missing modeling of their actual localization. For example, we did not include endosomal and actin cytoskeleton marker proteins within the training dataset. Therefore, proteins localizing to these compartments may be classified to another subcellular niche or labeled “unknown.” For example, well-known endosomal proteins like Vti1b or Vps4b may be found classified to the PM or the cytosol cluster within our data. In addition, as the ribosome and proteasome cluster do not represent membrane-bound niches but high-mass multiprotein complexes, there is the possibility of other protein complexes to show a similar fractionation pattern. Thus, other soluble multiprotein complexes may be classified to these compartments, as can be observed by ribosomal classification of transport protein particle (TRAPP) subunits in MPC-11 cells (Trappc1, Trappc2l, Trappc3, Trappc4, Trappc5, Trappc11, and Trappc12) in our data.

### Immunoglobulin Chains are Strongly Associated With ER Chaperones and Proteins Involved in Protein Glycosylation

Since we were interested in the antibody secretion process within both cell lines, we first determined the location of the antibody chains within both maps. We found both heavy and light antibody chains to be classified to the ER with high SVM scores (CHO-K1 heavy chain: 0.95, light chain: 0.87; MPC-11 heavy chain: 0.84, and light chain: 0.83). By calculating Pearson’s correlation coefficient between immunoglobulin G chains and all other proteins classified to the ER cluster, we estimated which proteins most likely were colocalized to the antibody chains (Table 1). In both cell lines, most of these proteins were associated with protein folding, protein glycosylation, oligosaccharyl transferase complex and protein translocation/transport in the ER. In addition, both cell lines showed proteins involved in cell migration/cell proliferation (Fgf18, Igfbp4, and Fndc3b in CHO-K1/Ccdc134; Fndc3b in MPC-11) and in lipid and cholesterol synthesis (Srebf1 in CHO-K1/Sc5d in MPC-11). In total, 28 of 40 proteins were found in both cell lines, with six proteins (Asph, Dnajc10, Fndc3b, Kdelc1, Ddost, and Rpn1) common among the highest correlating proteins to IgG chains. All proteins were localized to the ER in both cell lines, with the exception of Magt1 and Chpf showing differential localization in CHO-K1 cells (Magt1: unknown; Chpf: Golgi). Protein expression data from whole-cell lysates revealed that most of these proteins were highly expressed in MPC-11, whereas only three proteins (Dnajc10, Plod1, and Sep15) were elevated in CHO-K1 cells (Table 1).

**Table 1:** Top 20 proteins correlating highest with localization of antibody chains in spatial proteome maps of CHO-K1 and MPC-11 cells

CHO-K1			MPC-11		
Gene symbol	Function	Log2 fold change (CHO/MPC-11)	Gene symbol	Function	Log2 fold change (CHO/MPC-11)
*Asph*	Hydroxylation of EGF-like domains; calcium homeostasis	−0.62	*Asph*	Hydroxylation of EGF-like domains; calcium homeostasis	−0.62
*Dnajc10*	Protein disulfide reductase; protein folding; ERAD	4.64	*Dnajc10*	Protein disulfide reductase; protein folding; ERAD	4.64
*Fndc3b*	Regulation of adipogenesis	−0.25	*Fndc3b*	Regulation of adipogenesis	−0.25
*Kdelc1*	Protein-O-glycosylation	NA	*Kdelc1*	Protein-O-glycosylation	NA
*Ddost*	Protein-N-glycosylation; OST complex	−1.1	*Ddost*	Protein-N-glycosylation; OST complex	−1.1
*Rpn1*	Protein-N-glycosylation; OST complex	−1.05	*Rpn1*	Protein-N-glycosylation; OST complex	−1.05
*Ncln*	Ribosome–ER translocon	−1.28	*Sec61a1*	Ribosome–ER translocon	−1.26
*Nomo1*	Ribosome–ER translocon	−0.84	*Sec63*	Ribosome–ER translocon; protein trafficking within ER	−0.91
*Dnajb11*	Protein folding in ER; ERAD	−2.6	*Tbl2*	UPR; response to hypoxia; stress response	−0.01
*Uggt2*	Protein glycosylation; quality control in ER	NA	*Magt1*	Protein-N-glycosylation; OST complex	NA
*Poglut1*	Protein-O-glycosylation	NA	*Krtcap2*	Protein-N-glycosylation; OST complex	NA
*Ppib*	Peptidyl-prolyl *cis*–*trans* isomerase; protein folding	−1.06	*Mlec*	Protein-N-glycosylation	NA
*Clip3*	Trans-Golgi network–endosome trafficking	NA	*Sep15*	ER quality control	2.50
*Atp13a1*	Mitochondrial protein translocation from ER to mitochondria	−2.15	*Ssr4*	Ribosome–ER translocon; calcium homeostasis	−2.28
*Fgf18*	Cell proliferation; cell differentiation; and migration	NA	*Tram1*	Ribosome–ER translocon	NA
*Srebf1*	Cholesterol biosynthesis	NA	*Sc5d*	Cholesterol biosynthesis	NA
*Igfbp4*	Insulin-like growth factor signalling	NA	*Ccdc134*	ERK, JNK signaling	NA
*Mtdh*	NF-κB signaling; transcriptional coactivator	NA	*Chid1*	Saccharide binding, LPS binding; innate immune response	−0.15
*Mydgf*	MAPK signaling	−1.13	*Plod1*	Hydroxylation of lysine residues in collagen	1.92
*Ogfod3*	Oxidoreductase	NA	*Chpf*	Glycosylation of chondroitin	NA

Abbreviations: EGF, epidermal growth factor; ERAD, endoplasmic reticulum–associated protein degradation; LPS, lipopoysaccharide; MAPK, mitogen-activated protein kinase; NA, not available; OST, oligosaccharyl transferase.

The proteins are involved in highly relevant molecular processes in cellular antibody generation and release.

### Protein Localization Differences Across Spatial Maps Reveal Differentially Regulated Pathways

After ensuring quality of subcellular resolution for both datasets, we were able to analyze the datasets for significant DL proteins. As previously established methods for protein translocation detection in profiling spatial proteomics were developed and applied to analyze translocations within one cell system upon treatment (30, 52, 53, 54), most of these methods require that the majority of protein fractionation profiles do not change between conditions. Comparison of organelle fractionation profiles between the datasets reported here showed systematic shifts of organelle profiles, for example, Golgi apparatus, mitochondrion, and peroxisome (supplemental Fig. S6). Therefore, we reformulated the analysis as detection of significant changes in protein-to-cluster distances by expressing the relative protein position as a 10 value distance profile to each cluster. Translocation direction then was assessed by determining the closest clusters for each protein within each map. We determined a protein as DL if both the distances to the closest clusters were statistically significant (two-sided Student’s *t* test, BH-FDR = 0.05). To this dataset, we added proteins, which already were classified to different subcellular compartments in both cell lines, yielding 61 DL proteins in total (supplemental Table S5).

**Fig. 3:**
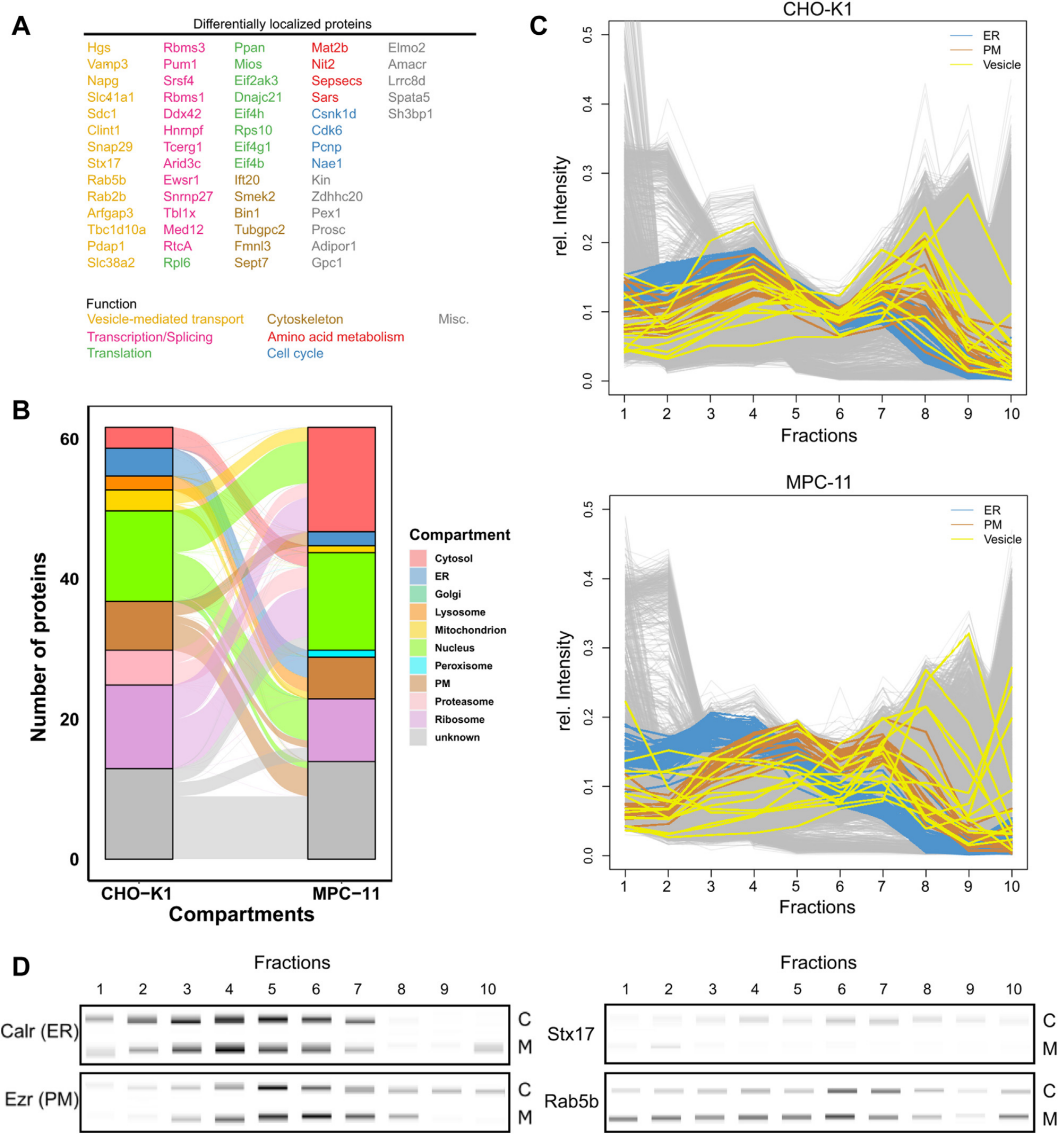
**Differentially localized (DL) proteins detected by multidimensional distance measurement.***A*, 61 proteins were identified as DL between CHO-K1 and MPC-11 cells. Proteins were ordered and colorized according to associated biological pathways. *B*, alluvial plot showing the number of DL proteins between CHO-K1 and MPC-11 cells distributed across subcellular compartments. Proteins labeled as unknown in both cell lines were determined as differentially located if they showed significant distance changes to the nearest compartment. *C*, protein profiles for DL proteins associated with vesicle-mediated transport in CHO-K1 (*above*) and MPC-11 (*below*) cells. ER and PM marker proteins are indicated in *blue* and *brown*, respectively. Vesicle-mediated transport–associated proteins are indicated in *yellow*. *D*, multiplexed Western blot validation of protein profiles for selected proteins. DigiWest results for ER (Calr) and PM (Ezr) marker proteins are indicated as well as for Stx17 and Rab5b. C and M letters on the *right* indicate the bands in CHO-K1 and MPC-11 cells, respectively. CHO, Chinese hamster ovary; ER, endoplasmic reticulum; MPC-11, Merwin plasma cell tumor-11; PM, plasma membrane.

Functional classification of DL proteins revealed a large portion of proteins to be associated with vesicle-mediated transport, transcription, and translation (Fig. 3*A*). A smaller proportion of proteins was associated with the cytoskeleton or PM, amino acid metabolism, and cell cycle. Qualitatively, most DL proteins were located to the nucleus, ribosome, and cytosol, with a smaller fraction of proteins located to either ER or PM (Fig. 3*B*). In the following, the functions of the largest protein groups are highlighted.

Proteins associated with vesicle-mediated transport mainly showed differential localization between organelles of the secretory pathway (Fig. 3*C*). Stx17, Rab2b, and Rab5b were classified to the PM in CHO-K1 cells, whereas they were localized near the ER and Golgi apparatus in MPC-11 cells. Differential localization for Stx17 and Rab5b was confirmed by multiplexed Western blotting (Fig. 3*D*). In addition, more vesicle trafficking–associated proteins (Clint1, Vamp3, Napg, and Arfgap3) showed movement toward the PM cluster in CHO-K1 cells compared with more dispersed distribution in MPC-11 cells. In contrast, Tbc1d10a and Sdc1 were classified to the ER in CHO-K1 and to the PM in MPC-11 cells.

**Fig. 4:**
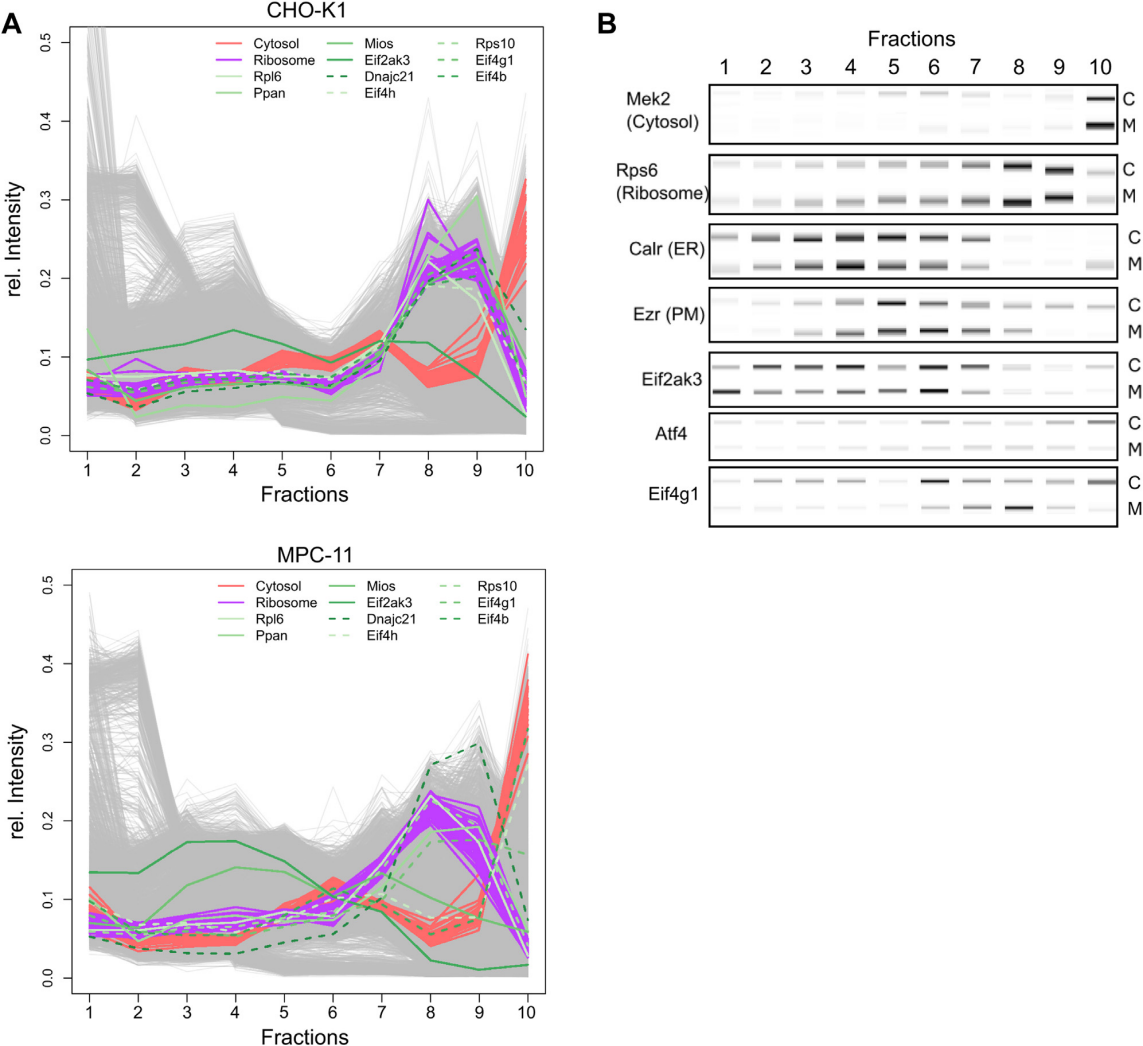
**Differentially localized proteins associated with translation.***A*, protein profiles of translation-associated differentially localized proteins for CHO-K1 (*above*) and MPC-11 cells (*below*). Translation-associated proteins are indicated in shades of *green* and distinct line types. Marker proteins for cytosol and ribosome are indicated in *red* and *purple*, respectively. *B*, validation of protein profiles quantified by multiplexed Western blot. Additional DigiWest results for marker proteins of the cytosol (Mek2), ribosome (Rps6), ER (Calr), and PM (Ezr) are indicated. The letters C and M on the *right* indicate the bands in CHO-K1 and MPC-11 cells, respectively. CHO, Chinese hamster ovary; ER, endoplasmic reticulum; MPC-11, Merwin plasma cell tumor-11; PM, plasma membrane.

Translation-associated proteins mainly were localized to ribosomes and nucleus in CHO-K1 cells, whereas in MPC-11 cells, they were distributed among cytosol, ribosome, and the nucleus (Fig. 4*A*). The translation initiation factors Eif4g1 and Eif4b were located at the ribosomes in CHO-K1 cells, whereas they were classified as nuclear (Eif4g1) and cytosolic (Eif4b) in MPC-11 cells. Eif2ak3 was classified to the PM in CHO-K1 cells and to the ER in MPC-11 cells. As Eif2ak3 is involved in UPR/induced stress response (ISR) signaling, we analyzed expression of the downstream effector Atf4 activity by multiplexed Western blotting (Fig. 4*B*). Atf4 expression was higher in CHO-K1 cells compared with MPC-11 cells. However, in both cell lines, Atf4 mainly was localized to the cytosol and thus unlikely to act in stress response, which would require nuclear localization. In addition, Eif2ak3 was found to mediate ER–PM contact sites in a Ca^2+^-dependent manner independently of UPR signaling (55). As a result, Stim1 and E-Syt1 relocated to the PM to form ER–PM contact sites to Orai1. Consistently, we found Stim1 and E-Syt1 to be colocalized with Eif2ak3 at the PM in CHO-K1 cells and at the ER in MPC-11 cells (supplemental Fig. S7).

Transcription factors (TFs) and RNA-binding proteins represented another large group of DL proteins. Here, nuclear localization of Tcerg1, Ewsr1, Tbl1x, and Med12 indicates transcription modulation in CHO-K1 cells, whereas the TFs Rbms1, Rbms3, and Arid3c were localized to the nucleus in MPC-11 cells (supplemental Fig. S8). Splicing-associated and mRNA-modifying proteins, Pum1, Ddx42, RtcA, Srsf4, Hnrnpf, and Snrnp27, were found distributed across ribosome, cytosol, and nucleus.

### Organelles of the Secretory Pathway Show Elevated Protein Masses in Myeloma Cells

In order to provide a more holistic approach for analyzing organelle composition across cell lines, we used the proteomic ruler approach (31), which already was deployed by Itzhak *et al*. (30, 46) to investigate the quantitative organelle composition of primary mouse neurons and HeLa cells. This approach enabled us to estimate protein copy numbers and organelle protein mass from TMT-labeled fractions. In addition, we compared these metrics to mean copy numbers from unfractionated whole-cell lysates in order to assess the usage of TMT-quantified fractions within the proteomic ruler method. Correlation was high for both CHO-K1 (Pearson’s correlation coefficient = 0.68) and MPC-11 (Pearson’s correlation coefficient = 0.72) cells with a slope around 1 indicating high agreement between whole-cell lysate and TMT-labeled fractions (supplemental Fig. S9).

**Fig. 5:**
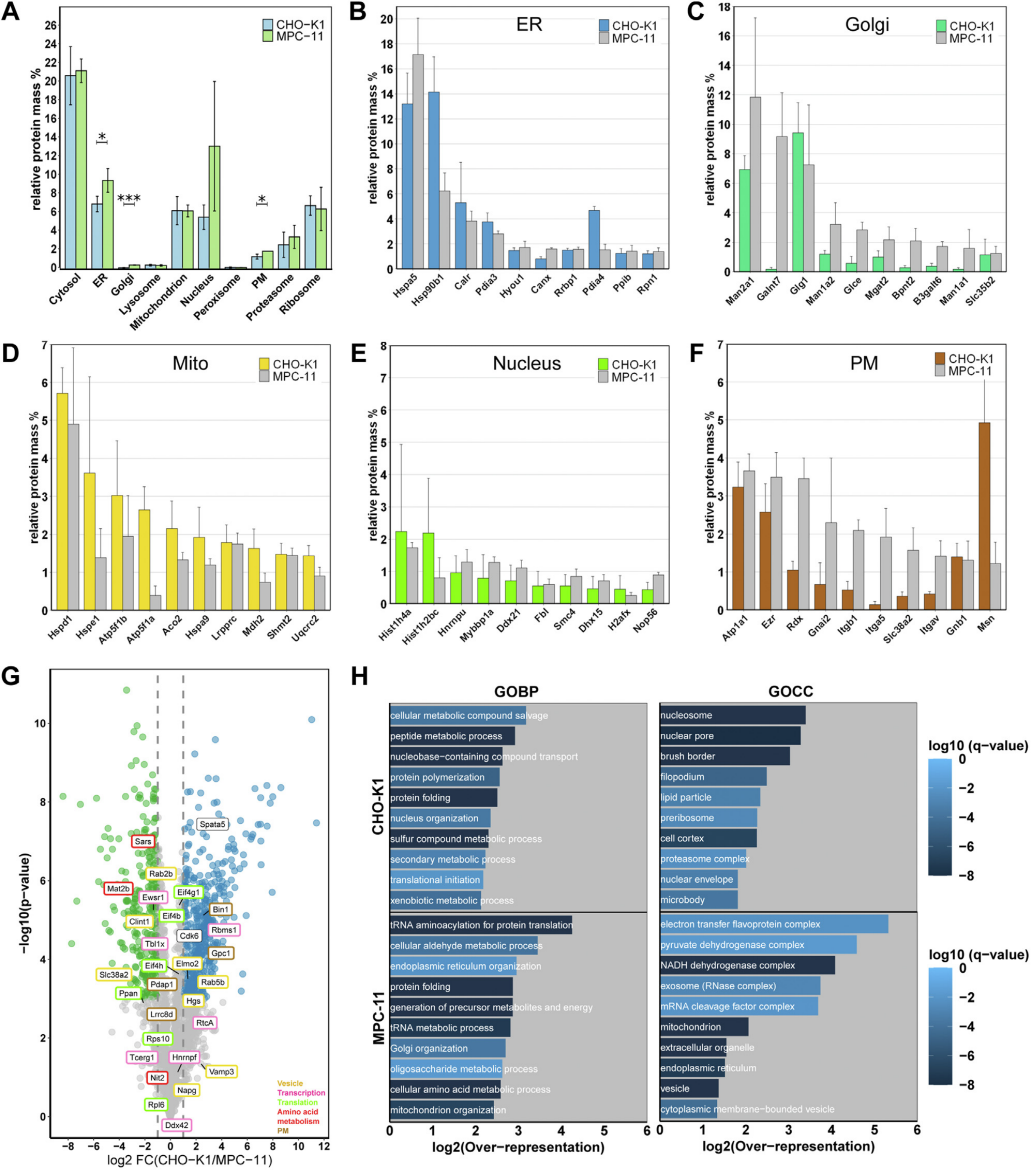
**Quantitative proteome representation of CHO-K1 and MPC-11 cells.***A*, proteomic ruler–derived relative organelle protein masses for CHO-K1 and MPC-11 cells. Statistical significance was calculated by Student’s *t* test (two-sided, *p* < 0.05). *B*–*F*, proteomic ruler–derived protein mass contribution to individual organelles of the top 10 contributing proteins for CHO-K1 (indicated in *color*) and MPC-11 (in *gray*). Bars represent mean values from three independent replicates with error bars indicating the standard deviation. *G*, volcano plot depicting log2 fold changes and –log10-transformed *p* values of differentially expressed (DE) proteins from unfractionated cell lysates determined by DEqMS analysis (BH-FDR = 0.05). Proteins significantly higher expressed in CHO-K1 cells are colored *blue*, whereas proteins higher expressed in MPC-11 cells are depicted in *green*, respectively. Labels represent differentially localized (DL) proteins found within the dataset. *H*, Gene Ontology (GO) enrichment analysis of DE proteins from unfractionated cell lysates of CHO-K1 and MPC-11 cells. DE proteins were annotated with GO terms and enrichment analysis (Fisher’s exact test, BH-FDR = 0.02) conducted against the complete mouse proteome (SwissProt, UP000000589). The top 10 enriched terms are depicted as bars with bar length according to the over-representation. Bar colors represent log10-transformed FDR-corrected *p* values. BH-FDR, Benjamini–Hochberg-corrected false discovery rate; CHO, Chinese hamster ovary; MPC-11, Merwin plasma cell tumor-11.

We subsequently combined proteomic ruler–derived protein copy numbers per cell and subcellular localization prediction to infer protein mass contributions to organelles (Fig. 5*A* and supplemental Table S6). Organelle protein mass was similar for most organelles between both cell lines. Hereby, the cytosol cluster represented the largest mass contribution in both cell lines (∼20% in both), followed by ER (7% in CHO, 9% in MPC-11), mitochondrion (∼6% in both), ribosome (∼6% in both), and nucleus (6% in CHO, 13% in MPC-11). MPC-11 cells showed higher protein mass in organelles of the secretory pathway (ER, Golgi, and PM, two-sided *t* test, *p* < 0.05). Investigating mass contributions of single proteins to their respective organelle revealed similar protein mass contribution for ER-classified proteins in both cell lines (Fig. 5*B*), indicating that these may be relevant for the general function of the ER and do not resemble specific adaptions toward higher protein secretion. Similarly, mitochondrion and nucleus composition did not differ much between cell lines (Fig. 5, *D* and *E*). However, composition of the Golgi apparatus and PM was strikingly different between both cell lines (Fig. 5, *C* and *F*). Golgi apparatus proteins involved in protein glycosylation (Man2a1, Man1a2, Man1a1, Mgat2, Galnt7, and Glce) showed higher mass contributions in MPC-11 cells. Proteins with highest mass contributions to the PM were represented by cytoskeleton-anchoring to the PM (Ezr, Rdx, and Msn) and cell–extracellular matrix (ECM) contact proteins (Itgb1, Itga5, and Itgav). For cytoskeleton-anchoring proteins, Msn represented the predominant protein in CHO-K1, Rdx was elevated in MPC-11, and Ezr showed similar mass contribution in both cell lines. In MPC-11 cells, integrins (Itgb1, Itga5, and Itgav) were among the proteins with highest mass contribution and thus showed higher expression compared with CHO-K1 cells.

Finally, we included MS data from unfractionated cell lysates of both cell lines in order to provide an independent dataset to assess protein abundance and DE proteins. Notably, to directly compare abundances of mouse and hamster proteins, we used an artificially generated database for MS data search, containing only sequence-identical peptides between both species. This restricted the number of quantified proteins across all six replicates to 2137 (supplemental Table S7).

To investigate if expression differences affect subcellular localization of proteins, DL proteins were mapped to the whole lysate dataset, where differential expression between CHO-K1 and MPC-11 cells was determined (DEqMS analysis, BH-FDR < 0.05). In general, DL and DE proteins did not correlate (Fig. 5*G*). Consequently, for DL proteins found to not be differentially expressed, we could conclude that differential localization was achieved independently from expression, for example, by protein modification or active protein transport *via* interaction partners. This difference in localization without differential expression in the two cell lines was found for 11 proteins: Eif4h, Lrrc8d, Hgs, RtcA, Rps10, Tcerg1, Hnrnpf, Vamp3, Napg, Rpl6, and Ddx42. All other DL proteins within the dataset were found to be DE proteins between CHO-K1 and MPC-11 cells. Here, affection of localization by expression differences or vice versa could not be ruled out and needs further examination.

DE proteins were analyzed by GO enrichment analysis and revealed proteins associated with nucleosomes, nuclear pores, cell cortex, and the proteasome to be enriched in CHO-K1, whereas in MPC-11 cells, proteins associated with tRNA-, ER-, Golgi apparatus-, mitochondria-, and oligosaccharide metabolic process were enriched (Fig. 5*H*). This was in accordance with elevated ER and Golgi protein mass in MPC-11 cells obtained by the proteomic ruler. For example, we found the Golgi resident proteins Man2a1 and Mgat2 to be higher expressed in MPC-11 cells, as predicted by proteomic ruler. In addition, mitochondria-associated proteins also were enriched in MPC-11 cells. Although the proteomic ruler quantified less nuclear protein mass, nucleosome and nuclear pore proteins were enriched in CHO-K1 cells.

## Discussion

We successfully applied a well-established workflow for subcellular proteomics analysis in the form of a modified LOPIT–DC protocol to two antibody-secreting cell lines for which the spatial distribution of a major part of the proteome hitherto was unknown. Here, we report the subcellular localization of 2444 proteins in CHO-K1 and 2577 in MPC-11 cells. Validation of machine learning results by multiplexed Western blotting as well as comparison to pre-existing datasets and databases revealed high accuracy of subcellular protein localization prediction. Furthermore, we combined protein localization with protein expression data and the proteomic ruler workflow to elucidate the organelle architecture and to show distinct differences in secretory organelles between both antibody-secreting cell lines.

The marker set deployed in this study was based partially on a proposed general organelle marker set (42) and proteins already used in spatial proteomics workflows (29). Successful application of this marker set to two cell types (PCD and CHO cells) as well as one organism (*C. griseus*) not yet analyzed in a profiling spatial proteomics workflow further validate the proteins to be used as a general organelle marker set for these type of experiments.

For well-resolved subnuclear compartments, the hyperLOPIT workflow includes a chromatin preparation step prior to centrifugation-based fractionation (29). Despite a missing chromatin preparation step in the LOPIT–DC workflow, a semisupervised Bayesian novelty detection algorithm resolved a nuclear chromatin and a nuclear nonchromatin cluster when applied to a LOPIT–DC dataset (56), indicating that subnuclear resolution can be achieved by the workflow. Our CHO-K1 data reported here further support this approach, whereas in MPC-11 cells, subnuclear resolution was not achieved. A reason for the higher subnuclear resolution in CHO-K1 may be that MPC-11 cells commonly show nucleus fragmentation, which may affect the fractionation of chromatin and nonchromatin nuclear proteins.

Benchmarking of the protein classifications to previously published datasets and databases (32, 47, 48) yielded highly according results for all membrane-bound organelles, showing the biological accuracy of our data (supplemental Figs. S4 and S5). For ribosomal, proteasomal and cytosolic classifications, comparison results were ambiguous as many proteins from these clusters showed nuclear annotations in databases. Nuclear annotations of proteins classified to the cytosol stemmed only from GOCC annotations and mostly represented proteins shuttling between nucleus and cytosol (exportins, importins, and nuclear carrier proteins). Ribosomal proteins often show ambiguous subcellular annotations as they are associated to the ribosomal compartment as well as to the nucleolus and hence the nucleus. In addition, of the two databases used for comparison of our data, only GOCC included information about ribosomal and proteasomal proteins.

Our data showed that in both cell lines, immunoglobulin heavy and light chain localized to the ER and showed colocalization to proteins mainly involved in protein folding and glycosylation (Table 1). Elevated expression of nearly all these proteins in MPC-11 cells indicated increased protein glycosylation and folding capacity and thus a further adapted ER environment in MPC-11 cells. Concordantly, protein folding was found to be enriched in murine SP2/0 cells compared with M-CHO cells (57), further supporting our results that protein folding seems to be elevated in a PCD phenotype.

It should be emphasized, that profiling spatial proteomics results are not sufficient to prove actual protein–protein interactions (42). However, determination of direct interaction partners of the recombinant proteins within the ER may be of great interest, as they further could elucidate, that the cellular environment recombinant proteins have to traverse to be secreted eventually. Among the colocalizing proteins, Magt1 and Chpf displayed different subcellular localizations. The multifunctional protein Magt1 is an important component of the Stt3b-containing oligosaccharyl transferase complex and therefore involved in protein glycosylation (58, 59). However, a putative function as Mg^2+^ transporter at the PM in epithelial cells also is indicated (60), which might explain the mixed fractionation profile between ER and PM in CHO-K1 cells. Chpf is involved in ECM synthesis at the Golgi apparatus, but different isoforms may also locate to mitochondria (61). An ER localization was not reported previously. As CHO cells are reported to synthesize higher amounts of undesired ECM (62), Chpf may represent a knockdown/knockout target for cell line engineering.

We successfully applied a reformulated workflow for the detection of DL proteins as multidistance measurement. As Crook *et al*. (56) noted, protein translocation analysis by distance measurements needs subcellular context, that is, information between which organelles the translocation occurs. By inferring the closest cluster for each protein and linking this information to the statistical analysis, we provided subcellular context to our analysis. Moreover, this workflow enabled us to use the full subcellular information obtained by all replicate measurements, whereas with, for example, magnetic resonance analysis, we only would have been able to use 32% of the obtained data (2958 proteins mapped between both maps of 5027 and 4102 proteins, respectively). However, we note that this approach may be biased by cluster size, as Mahalanobis distances will be smaller to a subcellular compartment with a wide distribution of data points compared to one with a smaller spread of data points. Thus, large clusters may “overlay” smaller clusters in near proximity. In our data, this may be observed by proteins classified to the proteasome or ribosome, but showing the nucleus as closest cluster as determined by the minimum Mahalanobis distance approach (*e.g.*, Fam133b, Polr3c). Thus, our ability to detect proteins moving toward/from the proteasome or ribosome in one of the cell lines, might be hampered.

### Vesicle-Mediated Transport

DL analysis identified vesicle-mediated transport, translation, and transcription as the main regulated pathways (Fig. 3*A*). Proteins associated with vesicle-mediated transport consisted, for example, of proteins of the Rab family, SNAREs and clathrin-associated proteins. The small GTPases Rab2b and Rab5b are required for different stages of vesicle trafficking between the organelles of the secretory pathway. Whereas Rab2b travels between ER and Golgi apparatus, Rab5b may be involved in vesicle transport between endosomes and the PM (63). Several omics studies in CHO cells showed elevated expression of Rab and other vesicle-associated proteins in cell lines with high productivity (13, 23, 64). We found that expression of Rab2b increased in MPC-11 cells, whereas Rab5b was highly expressed in CHO-K1 cells (Fig. 5*G*), indicating different parts of vesicle traffic to be differentially regulated in both cell lines. Rab2b, and therefore the ER to Golgi transport, thus represents an additional target for overexpression in CHO cell engineering.

Stx17 is a SNARE protein, which is involved in autophagy by mediating fusion of lysosomes with autophagosomes as well as maintaining ER–Golgi intermediate compartment morphology (65). Snap29 also is involved in autophagy regulation and vesicle fusion at the PM, as depletion of this protein shows decreased PM fusion events (65). Both proteins either were classified to the PM (Stx17) or were located in close proximity to it (Snap29), indicating increased vesicle movement toward the PM in CHO-K1 cells. In MPC-11 cells, Stx17 was located near the ER and Golgi apparatus cluster, indicating that Stx17 mainly functions in maintaining ER–Golgi intermediate compartment structure. Previously, higher expression of Stx17 was found to correlate with higher recombinant protein production in CHO cells (23).

Tbc1d10a represents another vesicle-associated protein, which is involved in retrograde transport from the PM (66). Here, ER localization in CHO-K1 cells may indicate accumulation at the destination, whereas in MPC-11 cells, the protein accumulated at the start location. The heparan sulfate proteoglycan Sdc1 usually is located at the PM acting as linker between the cytoskeleton and ECM. However, Golgi localization also was confirmed for this protein where it mediates sorting of lipoprotein lipase (67). Localization at the ER was not reported for this protein, indicating that the main localization in CHO-K1 may be transient or hinting at another, yet unreported function of Sdc1 at the ER.

In summary, closer proximity to the PM in CHO-K1 cell compared with MPC-11 of a large portion of these proteins indicated an accumulation at the CHO PM, which in turn may affect long-term protein secretion. Therefore, recycling and retrograde vesicle proteins may represent a possible target for production cell engineering. Analysis of the membranome (vesicle-bound proteins) in M-CHO cells reported a depletion of secretory pathway and membrane proteins (57). However, we found no evidence for such a depletion. Thus, further investigation of the secretory vesicles in various CHO cell lines may be required for a proper elucidation of vesicle trafficking.

### Translation

Proteins associated with translation consisted of ribosomal proteins (Rpl6 and Rps10), proteins involved in ribosomal biogenesis (Ppan and Dnajc21), and components of the eukaryotic translation initiation factor 4F (eIF4) complex (Eif4b, Eif4g1, and Eif4h). Mios, which functions as part of the GATOR2 complex within the amino acid–sensing branch regulation of mTOR complex 1 (72) and the UPR/ISR sensor Eif2ak3 displayed differential localization as well. The eIF4 complex is required for cap-dependent translation by binding both mRNA and ribosomes and thus brings them in close proximity (68). Eif4b binds to Eif4a and Eif3a, allowing interaction between both translation complexes. Eif4g1 acts as scaffold protein and mediates binding of the other eIF4 subunits to form the eIF4 complex, whereas Eif4h functions as regulatory subunit in order to enhance Eif4a activity. The close proximity to the ribosome cluster of all three subunits indicates elevated involvement in translation in CHO-K1 cells, whereas in MPC-11 cells, the proteins were found in the cytosol (Eif4b) or the nucleus (Eif4g1).

Usually ER resident, Eif2ak3, mediates the UPR and ISR by phosphorylation of Eif2s1 (also known as eIF2alpha), thus causing global arrest of cap-dependent translation at the ribosome and elevated translation of cap-independent transcripts such as Atf4 or Atf5, both downstream mediators of the stress response (69). Close proximity to ER–PM contact site mediating proteins Stim1, E-Syt1, and Orai1 in CHO-K1 cells may indicate its involvement in regulating Ca^2+^ homeostasis within the cell and ER (55). As a consequence of Ca^2+^ depletion in ER, ER–PM sites form and allow Ca^2+^ replenishment as well as lipid transport across both compartments (70). Subsequently, prolonged Ca^2+^ dysregulation may lead to decreased protein folding capacity within the ER and to enhanced ER stress signaling (71). As Atf4 was not located to the nucleus (Fig. 4*B*), there was no sign of prolonged stress signaling in both cells. Calcium signaling also was found enriched in the transcriptome and proteome of SP2/0 cells in comparison to M-CHO cells, supporting the results reported here (57). As Ca^2+^ signaling and homeostasis previously was not thoroughly investigated in CHO cells so far, this indicates another possible pathway to be addressed in cell line engineering.

For the GATOR2 subunit Mios, we determined proximity to the nucleus and ribosome in CHO-K1 cells, whereas it was located close to the PM cluster in MPC-11 cells. At the lysosome, GATOR2 functions as activator of mTOR complex 1 and subsequently increases protein translation if amino acid abundancy is high (72). In addition, cytosolic and nuclear localization of Mios were reported (26), but protein functions at these sites need to be elucidated yet.

### Transcription

Differential subcellular distribution of proteins involved in transcription regulation (supplemental Fig. S8) (Ewsr1, Tbl1x, Rbms1, Rbms3, Tcerg1, Med12, and Arid3c) indicated differentially regulated gene expression between both cell lines. Tcerg1, Ewsr1, Tbl1x, and Med12 were found localized to the nucleus in CHO-K1 and thus may actively function as transcription modulators here, whereas, this was the case for Rbms1, Rbms3, and Arid3c in MPC-11 cells. Ewsr1, a transcriptional repressor affecting multiple cellular pathways, such as mitochondrial function (73) and autophagy (74), may also be involved in enhanced collagen production (75). Especially the last function may be interesting, since CHO cell lines are known to secrete large amounts of undesired ECM-associated proteins, for example, collagens, reflecting possible remnants of their epithelial origin (62). Further investigation of the role of Ewsr1 in CHO cells may evaluate its benefit in production cell engineering. Med12, Tbl1x, and Tcerg1 function as general transcription modulators with contrary implications as Med12 and Tbl1x activate, whereas Tcerg1 inhibits transcription. The TFs with nuclear localization in MPC-11 cells consisted of Rbms1 and Rbms3, modulators of Myc and Wnt regulation. Transcriptional modulation of Myc could be linked to the cancerous phenotype of MPC-11 cells. Negative regulation of Wnt pathway signaling by Rbms3 would match reported downregulation of the Wnt pathway during B-cell differentiation (76, 77). Since both Rbms1 and Rbms3 contain a RNA-binding domain, binding to rRNA and thus localization to ribosomes might occur. However, ribosomal localization was not reported previously. Finally, the Arid3a interactor Arid3c also was classified to the nucleus in MPC-11. By binding to Arid3a, Arid3c enhances Arid3a-dependent immunoglobulin H transcription in B cells (78). Although the cells analyzed here do not secrete immunoglobulin H-type antibodies, Arid3c localization to the nucleus indicated an involvement of these proteins in the secretion of additional types of immunoglobulins. Other transcription-associated DL proteins were found in splicing-associated proteins Srsf4, Hnrnpf, and Snrnp27 and RNA-modifying proteins RtcA, Pum1, and Ddx42. As these proteins showed no common trend to be localized at a certain compartment, further data are required to infer biological knowledge. Interaction with RNA might distribute these proteins across several compartments, such as nucleus, ribosome, and cytosol. Thus, colocalization to mRNA might present a way to investigate the function of these proteins.

The proteomic ruler method yielded higher relative protein organelle mass for secretory pathway organelles in MPC-11 cells, which was supported by enrichment of proteins associated to these organelles by DE analysis of whole-cell lysates (Fig. 5). Moreover, by inspecting protein mass contribution to single organelles, we found main differences in protein glycosylation–associated proteins within the Golgi apparatus with higher expression in MPC-11 cells, highlighting protein glycosylation as differentially regulated pathway between both cell lines (Fig. 5*C*). Several studies in CHO cells showed correlation between elevated expression of glycosylation-associated proteins and high recombinant protein expression (79), indicating that overexpression of these proteins improves recombinant protein expression. Also, PM proteins contributing to cytoskeleton anchoring showed different relative protein masses, which could be of interest for further investigation in both cell lines.

In order to prepare for high antibody secretion, B cells undergo massive changes in protein expression, especially enlarging the ER and increasing protein levels of, for example, chaperones *via* UPR (16, 80, 81). The ER and secretory pathway in CHO-K1 cells already were studied extensively, revealing impaired protein secretion mostly as consequence of missing folding capacity within the ER, resulting in insoluble protein agglomerates within the ER–Golgi intermediate compartment or ER itself (82, 83). Thus, several cell engineering studies targeted ER resident proteins as well as UPR-associated proteins for overexpression, with positive results for specific productivity (84, 85, 86).

Although mean nuclear protein mass was twice as high in MPC-11 cells, it showed high variance. We already expected elevated nuclear mass in these cells, as they show hyperdiploid to triploid chromosome numbers (49). Thus, heterogenic nucleus structures in this cells could explain the large variance in observed protein nuclear mass. Enriched nuclear pore proteins might indicate increased nucleus–cytosol trafficking in CHO-K1 cells. However, we did not find further support for increased nuclear export/import in either cell line. Further proteomic studies that focus on nuclear–cytosolic traffic may elucidate this process.

Furthermore, enriched mitochondrial proteins in MPC-11 cells emphasize energy metabolism as another pathway, which may represent adaption to high protein secretion. Here, proteomic comparison to naïve plasma cells could offer additional insights into energy supply for high protein secretion. Taken together, the high-resolution subcellular proteome data reported here represent an important addition to existing subcellular proteome datasets but more importantly also complement other large omics studies of CHO and myeloma cells with an additional layer of information, as the subcellular proteome organization of both cell types was not addressed before.

Because of their heavy employment in the pharmaceutical industry, CHO cells are well characterized by various omics studies aimed at improving recombinant protein production (2, 12, 14). However, despite such data enabled several successes in rational cell engineering on CHO cells, further improvements in CHO productivity should be possible, as natural plasma cells can show much higher productivity (87). The comparison of the subcellular proteomes of an antibody-secreting CHO-K1 cell line and a PCD myeloma cell line showed differences, which seem to be associated with antibody secretion. However, the results reported here further should be validated by cell engineering studies targeting the reported pathways and proteins. In the end, the data shown here may contribute to a better understanding of the cellular architecture and antibody secretion in both cell types and further improve CHO cell line productivity.

## Data Availability

The MS proteomics data have been deposited to the ProteomeXchange Consortium (http://proteomecentral.proteomexchange.org) *via* the PRIDE partner repository (88) with the dataset identifier PXD029115 and 10.6019/PXD029115. MSMS spectra were deposited at MS-Viewer (89) with search keys mh4zluopbr (MPC-11 replicate 1), eubymniai5 (MPC-11 replicate 2), bxfcnm1haz (MPC-11 replicate 3), 6v9pkdfqig (CHO-K1 replicate 1), dlx2ixr12g (CHO-K1 replicate 2), zqhdi6zysy (CHO-K1 replicate 3), and e2fbeqkqp (unfractionated cell lysates of CHO-K1 and MPC-11 cells).

## Supplemental data

This article contains supplemental data (Tables S1–S8 and Figs. S1–S9) (29, 30, 46).

## Conflict of interest

S. F. is an employee of Boehringer Ingelheim Pharma GmbH & Co KG, which develops and sells pharmaceuticals. All other authors declare no competing interests.

## Supplemental Data

Supplemental Data

TableS1

TableS2

TableS3

TableS4

TableS5

TableS6

TableS7

TableS8
