# Supplementary material for: Spatial Proteomics Reveals Differences in the Cellular Architecture of Antibody-Producing CHO and Plasma Cell–Derived Cells
Source: Mol Cell Proteomics. 2022 Aug 5;21(10):100278. doi: 10.1016/j.mcpro.2022.100278 (PMC9562429; doi:10.1016/j.mcpro.2022.100278)
Supplement: Supplemental Data [file mmc1.pdf]

# Spatial proteomics reveals differences in the cellular architecture of antibody-producing Chinese hamster ovary and plasma cell-derived cells

*Robin Kretz[1,2,3], Larissa Walter[1,2], Nadja Raab[4], Nikolas Zeh[4], Ralph Gauges[1], Kerstin Otte[4], Simon Fischer[5], Dieter Stoll[1,3]*

Affiliations

[1] University of Applied Sciences Albstadt-Sigmaringen, Department of Life Sciences, Anton-Guenther-Straße 51, 72488 Sigmaringen, Germany

[2] Biological Sciences, University of Constance, Universitaetsstraße 10, 78464 Konstanz, Germany

[3] NMI, Natural and Medical Sciences Institute at the University of Tuebingen, Markwiesenstraße 55, 72770 Reutlingen, Germany

[4] University of Applied Sciences Biberach, Institute of Applied Biotechnology, Hubertus-Liebrecht-Straße 35, 88400 Biberach, Germany

[5] Boehringer Ingelheim Pharma GmbH & Co KG, Bioprocess Development Biologicals, Cell Line Development, Birkendorfer Straße 65, 88397 Biberach, Germany

**Supplemental data:**

- **Fig. S1** Protein yield of fractions generated by differential centrifugation
- **Fig. S2** Spatial resolution of combined and replicate subcellular proteome maps
- **Fig. S3** Machine Learning performance of single replicate and combined maps
- **Fig. S4** Comparison of protein classification to Gene Ontology Cellular Component and UniProt Subcellular location databases
- **Fig. S5** Comparing protein classification to existing datasets
- **Fig. S6** Shifts in organelle marker protein fractionation profiles between CHO-K1 and MPC-11 cells
- **Fig. S7** Co-localization of ER-PM contact site proteins with Eif2ak3
- **Fig S8** Differential localization of transcription-associated proteins
- **Fig S9** Correlation of proteomic ruler-derived protein copy numbers

- **Table S1** Weighting factors for subcellular compartments
- **Table S2** organelle_markers.xlsx: Organelle marker proteins used to define subcellular compartments
- **Table S3** combined_maps.xlsx: Combined spatial maps of CHO-K1 and MPC-11 cells containing all proteins with subcellular classifications
- **Table S4** peptide_identifications.xlsx: Peptide identifications of single replicate maps and unfractionated cell lysates of CHO-K1 and MPC-11 cells
- **Table S5** DL_analysis.xlsx: DL-analysis results for 2958 matching proteins between CHO-K1 and MPC11
- **Table S6** Proteomic_ruler.xlsx: Results of the proteomic ruler for TMT-quantified protein fractions

- **Table S7** DE_proteins.xlsx: Results of Limma and DEqMS analysis of unfractionated cell lysates
- **Table S8** antibodies_DigiWest.xlsx: List of all antibodies used in DigiWest Assays

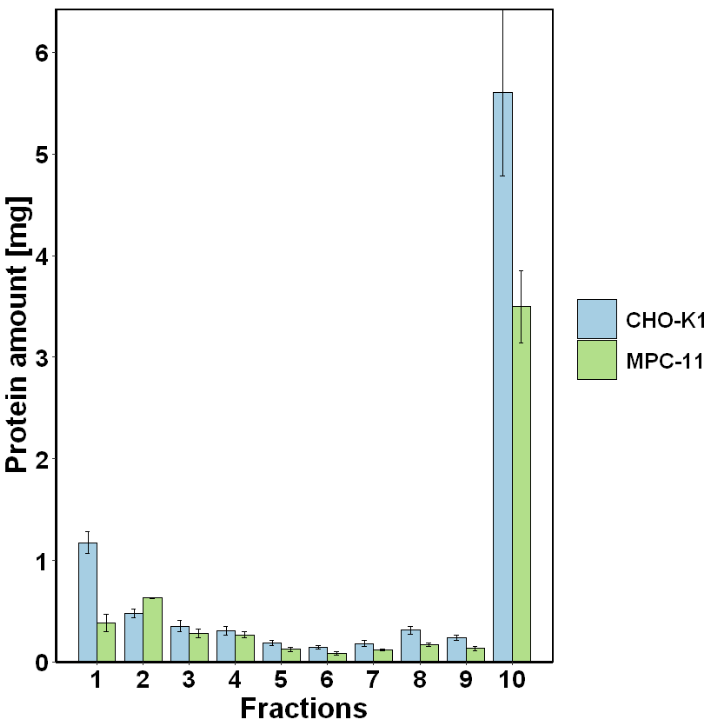


**Fig. S1** Total protein amount of fractions generated by differential centrifugation of CHO-K1 and MPC-11 cells. Protein amount was determined by BCA assay after lysis of organelles in the respective fraction. Mean ± sd protein yields from three independent replicates are shown for both CHO-K1 and MPC-11 cell lines.

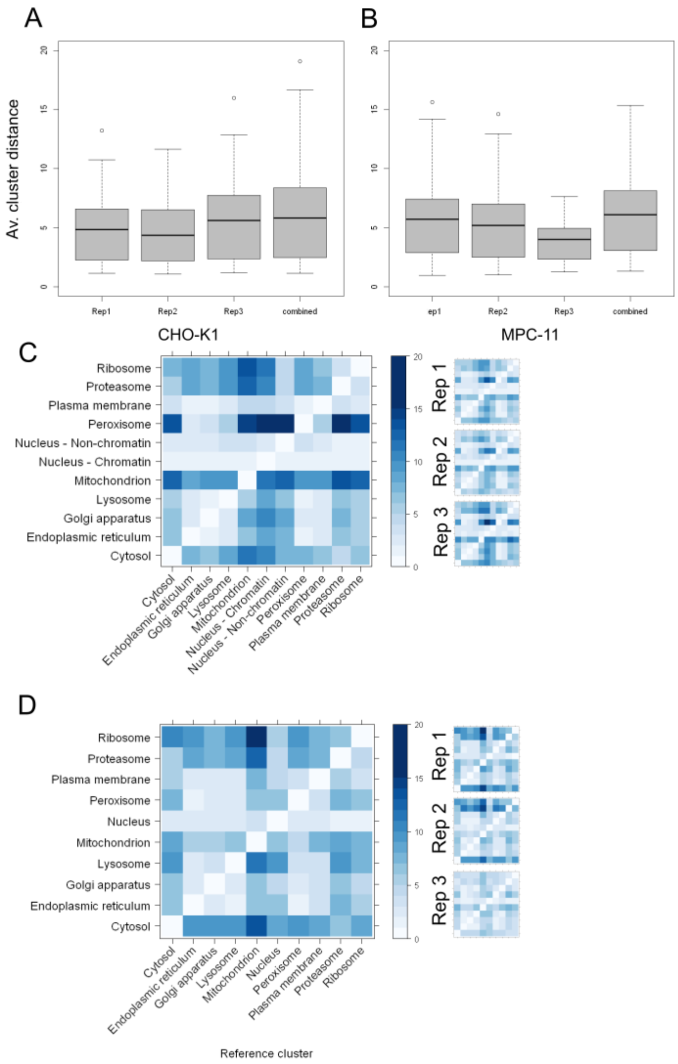


**Fig. S2** Spatial resolution of combined and replicate subcellular proteome maps. The distribution of average cluster distance between all organelle marker proteins for single replicate as well as combined maps are depicted as boy-whisker-plots for CHO-K1 (A) and MPC-11 (B) cells. Inter quartile range (IQR) is depicted as length of the box whereas whiskers are scaled to 1.5x IQR. The median is represented as line within the boxes. Median and maximal av. cluster distance increased slightly upon map combination in both cell lines. C) Normalized cluster distances as calculated by QSep of combined (large graph left) and single replicate maps for CHO-K1. Reference clusters are shown on the x-axis. Normalized QSep distances of single replicate maps are depicted in the three small graphs on the right side. D) Normalized cluster distances as calculated by QSep of combined (large graph left) and single replicate maps (small graphs on the right side) for MPC-11 cells. Reference clusters are shown on the x-axis.

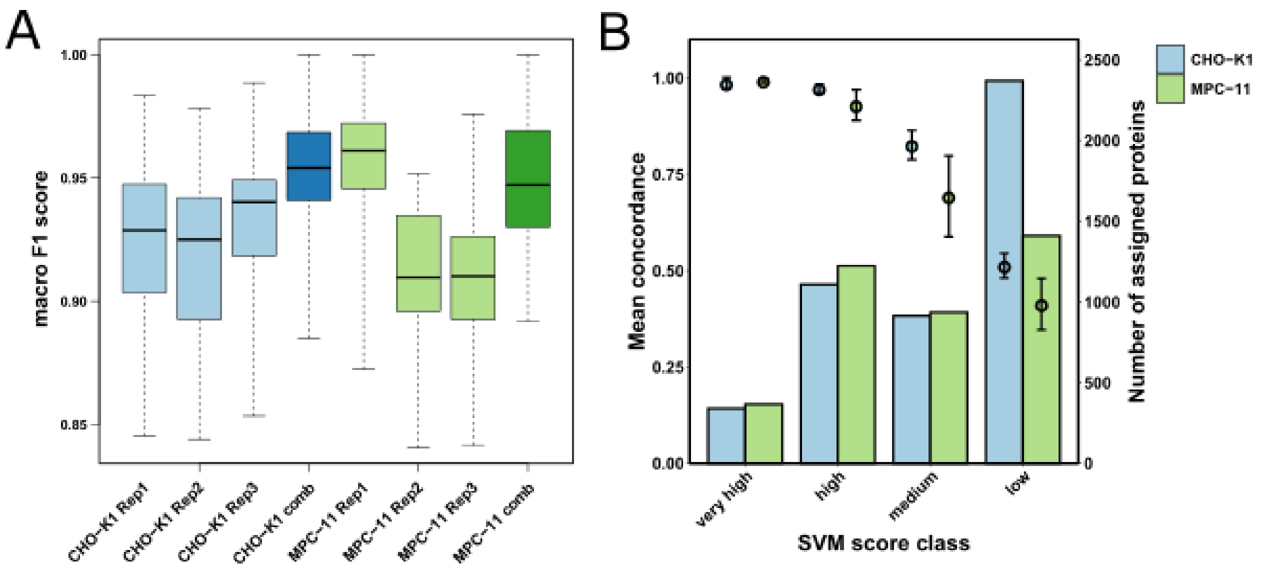


**Fig. S3** Machine Learning performance of single replicate and combined maps. A) Distribution of macro F1 scores (harmonic mean of recall and precision) for single replicate and combined maps after parameter optimization. Macro F1 scores were calculated after 100 round of 5-fold cross validation on organelle marker proteins. Lines within the boxes represent median values. Box length represent the inter quartile range and whiskers are scaled to maximum and minimum values, respectively. With the exception of MPC-11 replicate 1, combined maps showed higher performance as single replicate map. B) Pairwise single map concordance was calculated as fraction of identical protein classifications to all classifications within the respective SVM score class (very high (SVM score ≥ 0.9), high (SVM score 0.7 – 0.9), medium (SVM score 0.5 – 0.7) and low (SVM score < 0.5)). Depicted are mean concordances of all three pairwise map comparisons of one cell line. Error bars are scaled to standard deviation. The bars within the plot show the mean of all classified proteins within the SVM score class.

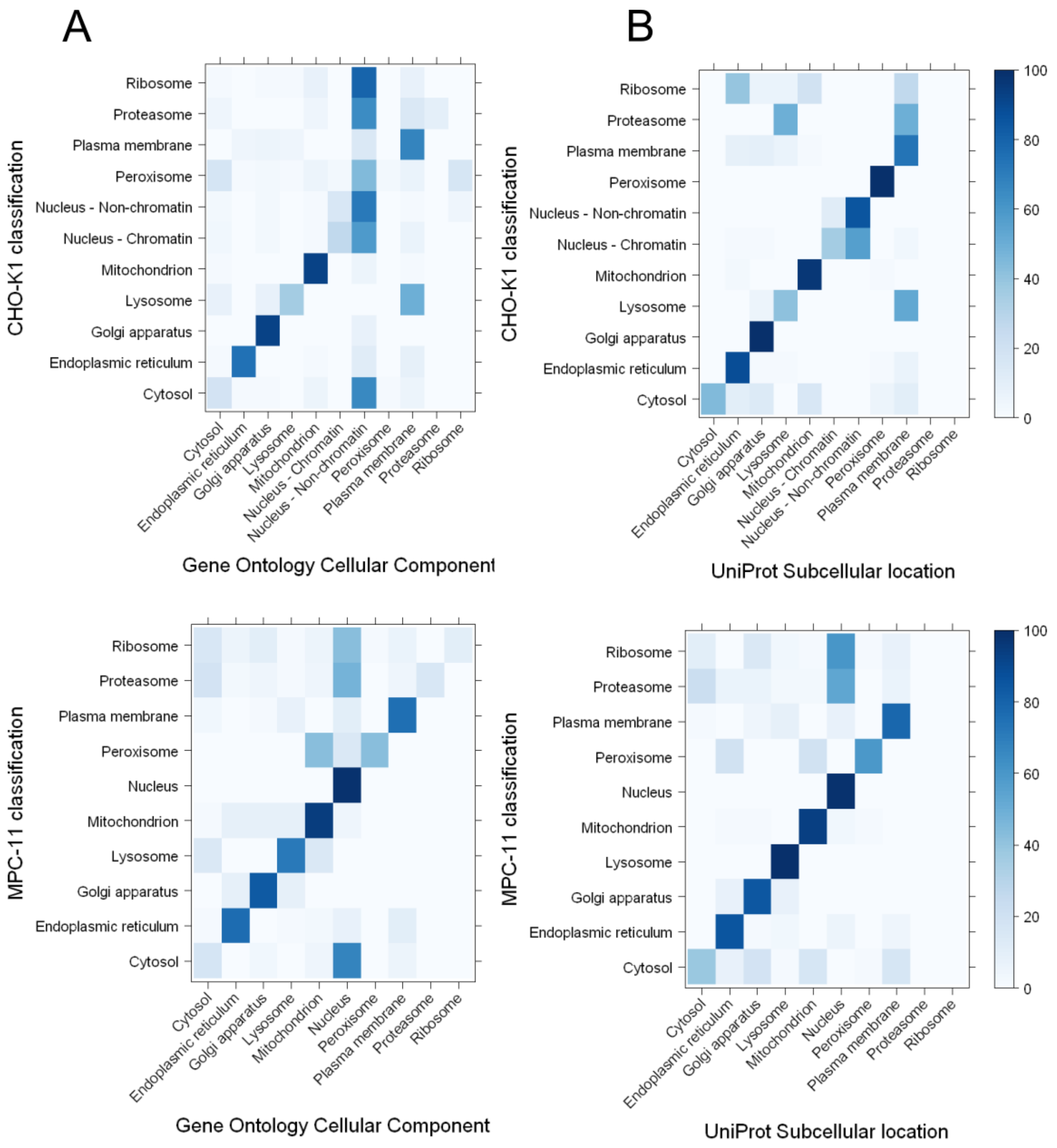


**Fig. S4** Comparison of protein classification to Gene Ontology Cellular Component and UniProt Subcellular location databases. A) Subcellular protein classification of CHO-K1 (above) and MPC-11 (below) cells were compared to Gene Ontology Cellular Component entries for the respective proteins. Colors represent the proportion of proteins annotated with a certain Gene Ontology/Subcellular location term to all proteins of a cluster. B) Concordance of subcellular protein classification to UniProt Subcellular location annotations. Colors represent the portion of similar classifications to all proteins classified to the respective cluster. Organelle marker proteins were removed prior to the analysis.

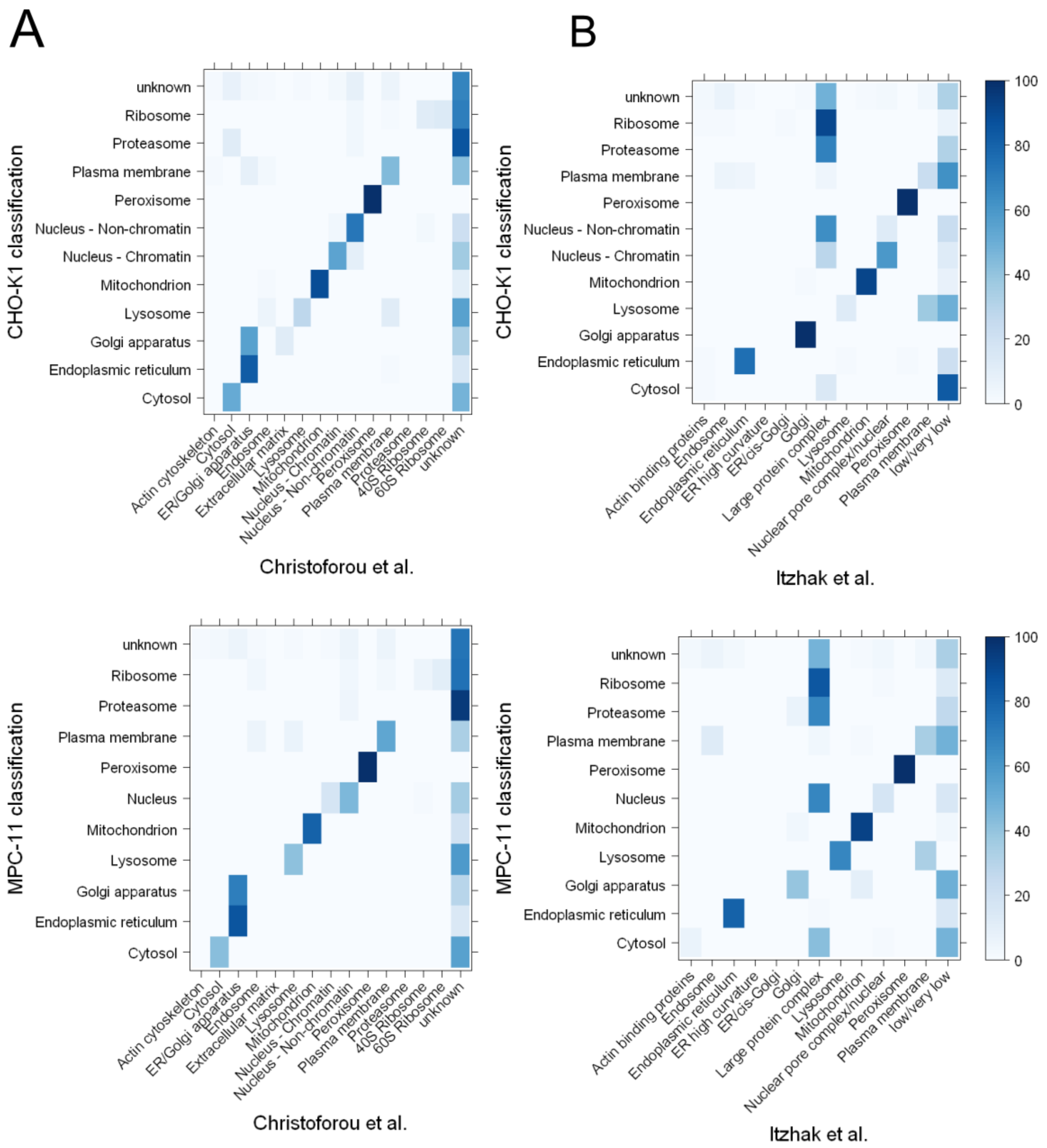


**Fig. S5** Comparing protein classification to existing datasets. Protein subcellular localization classification of CHO-K1 (above) and MPC-11 (below) cells were compared to classification of Christoforou *et al.* (1) (A) and Itzhak *et al*. (2) (B). Organelle marker proteins from all datasets were removed prior to comparison. Colors show the percentage intersection of proteins classified to both clusters. Itzhak *et al.* combined a large portion of nuclear, ribosomal and proteasomal proteins into a single cluster named 'Large protein compex'. Therefore correlation of our data to Christoforou *et al*. was higher.

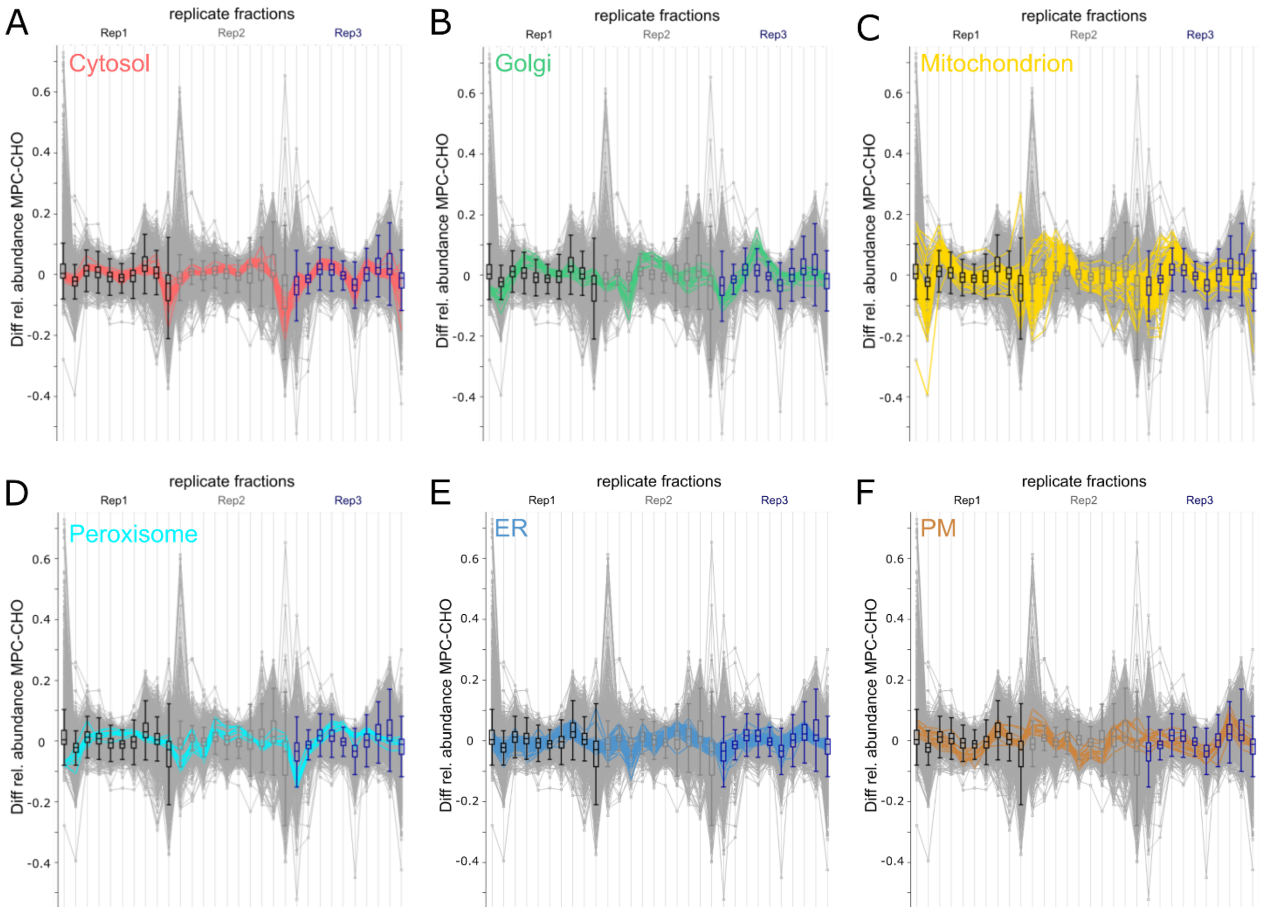


**Fig. S6** Shifts in organelle marker protein fractionation profiles between CHO-K1 and MPC-11 cells. Difference profiles for all 2925 mapped proteins between CHO-K1 and MPC-11 cells were calculated per replicate pair and the resulting fractionation profiles of organelle marker proteins displayed in color. Box whisker plots represent the distribution of all proteins within each difference fraction. Median values are represented by lines within each box and the IQR is depicted as box length. A) Cytosol marker proteins. B) Golgi apparatus marker proteins. C) Mitochondrion marker proteins. D) Peroxisome marker proteins. E-F) Fractionation profiles of organelle marker proteins with no apparent systematic shift E) ER marker proteins. F) PM marker proteins.

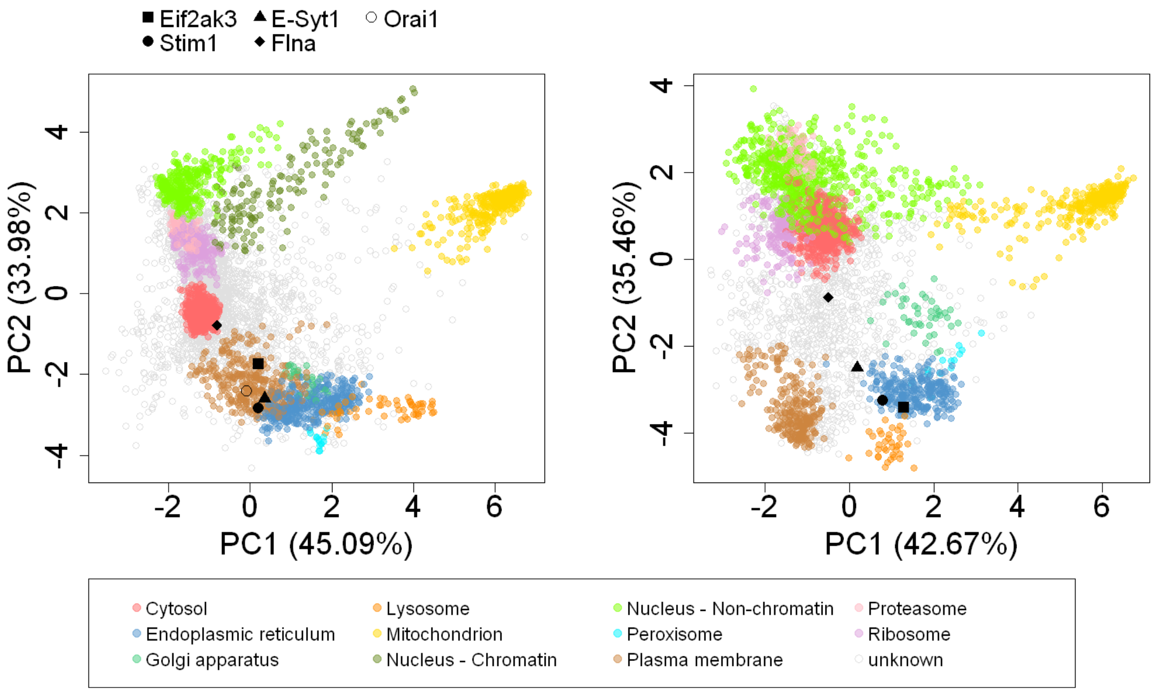


**Fig. S7** Co-localization of ER-PM contact site proteins with Eif2ak3. PCA plots of combined maps are colorized according to SVM classification with a threshold of SVM score ≥ 0.7. In CHO-K1 (left) cells, Eif2ak3 was predicted to be localized to the PM, together with mediators of ER-PM contact sites Stim1, E-Syt1 and Orai1. Flna, which causes Eif2ak3 dependent rearrangement of actin filaments necessary to mediate ER-PM contact sites was localized to the cytosol. In MPC-11 cells (right), Eif2ak3 was predicted to localize to the ER, together with Stim1. E-Syt1 was localized in proximity to the ER, whereas Flna was found close to the cytosol cluster. Orai1 was not detected in MPC-11 cells.

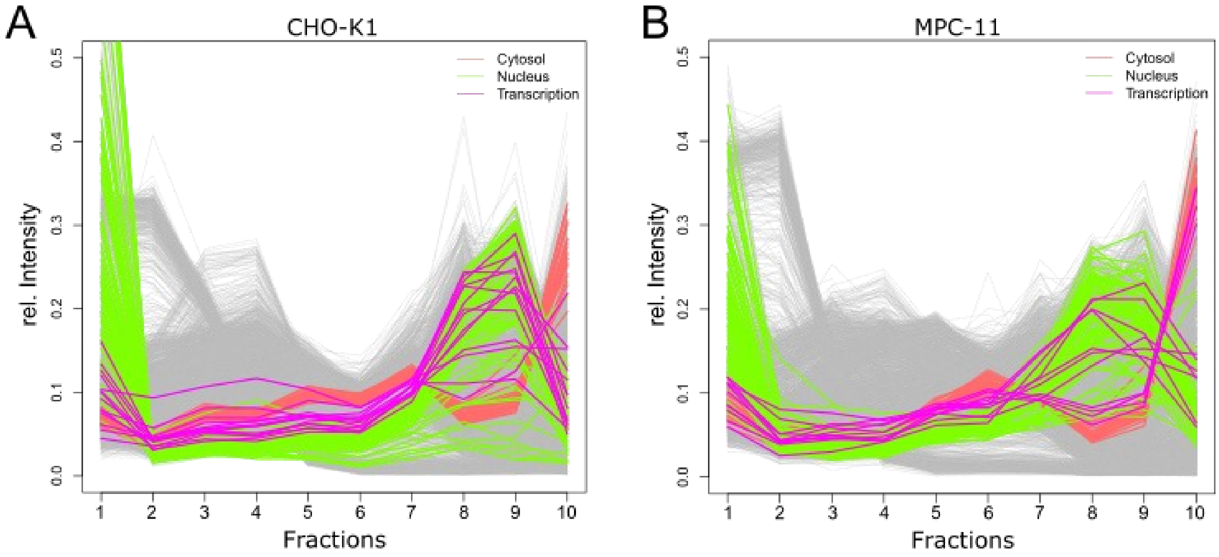


**Fig. S8** Differential localization of transcription-associated proteins. Fractionation profiles of CHO-K1 (left) and MPC-11 cells (right) for all quantified (grey), nuclear marker (green), cytosol marker (red) and differentially localized, transcription associated proteins (magenta).

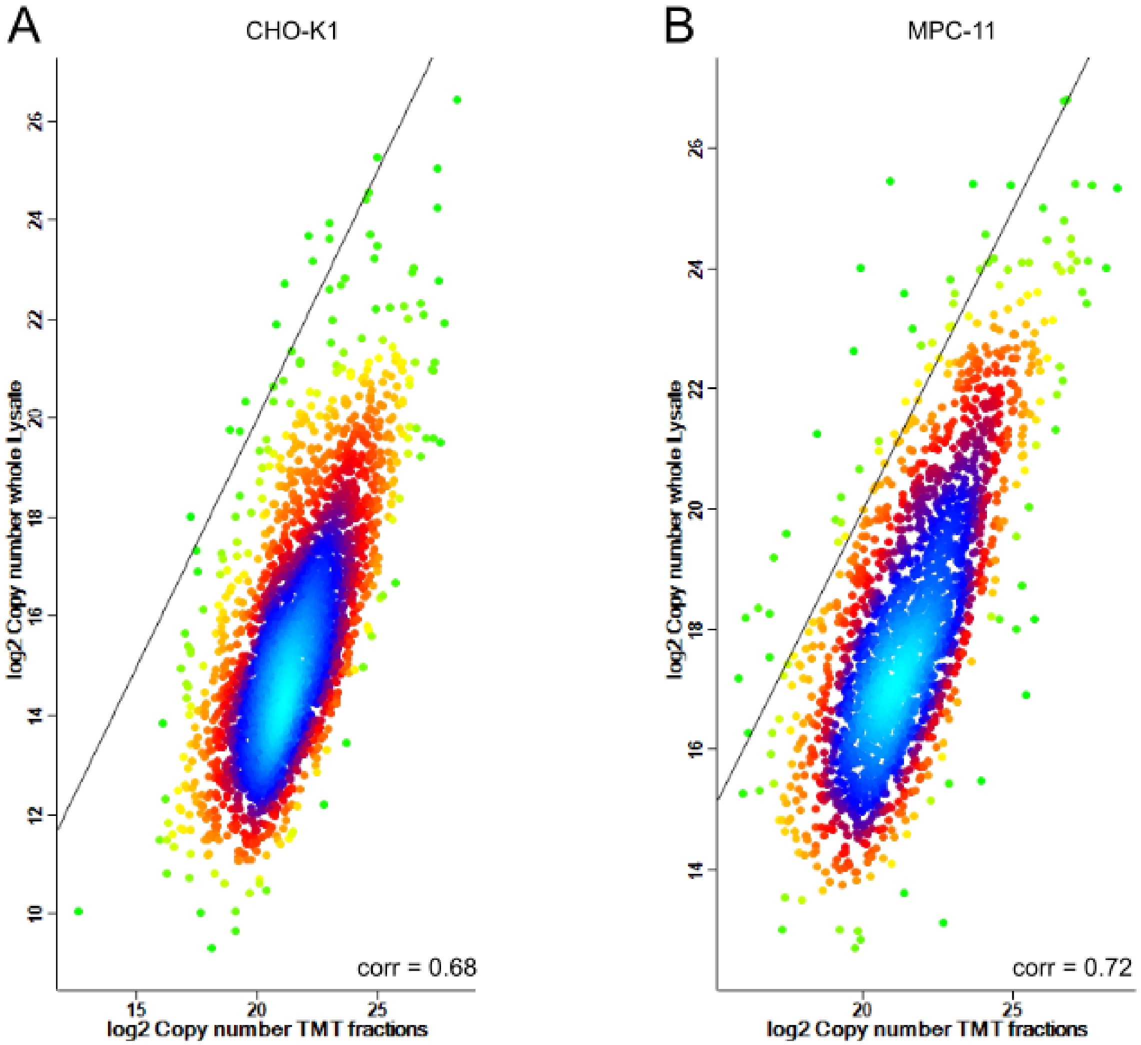


**Fig. S9** Correlation of proteomic ruler-derived protein copy numbers. Copy numbers from the proteomic ruler approach were calculated using two different data sets: the unfractionated lysates (whole lysate) and the fractionated, TMT-labeled data. TMT-labeled fractions were weighted according to fraction protein yield and summed up. Proteomic ruler-derived copy numbers were log2-transformed and depicted as scatterplot. Pearson's correlation coefficient was calculated for both cell lines. A) Correlation of log2-transformed copy numbers of CHO-K1 cells. B) Correlation of log2-transformed copy numbers of MPC-11 cells.

**Estimation of organelle protein masses**

Organelle protein masses were estimated by summation of protein mass/cell for all proteins classified to the respective subcellular compartment. Prior to summation, values were weighted according to their “nuclear”, “organelle” or “cytosolic” fractions. Different weighting factors (Table S1) were used, depending on the subcellular compartment and their fractionation profiles (Table S6). The selection of weighting factors mainly was based on the assumptions of Itzhak et al. 2016 (3) and Itzhak et al. 2017 (2), with few exceptions rooted in our observed data. Generally, we used the nuclear and organelle fraction as weighting factor for subcellular compartments of membrane-bound organelles. For ER and lysosome, we used a weighting factor of 1, since ER disruption by cell lysis causes lumenal ER proteins to leak into the cytosol. The same was done for lysosomes. Additionally, we observed a significant amount of nuclear proteins to display a cytosolic pool as well. These may arise from nucleus-cytosol shuttling proteins or from nuclear leakage during cell lysis. Therefore, we included the cytosolic fraction as well for nuclear proteins. Ribosomal proteins may be localized at the ER membrane, as cytosolic complexes or within the nucleus. Thus, we also set the weighting factor for ribosomes to 1. Lastly, cytosolic and proteasomal proteins were weighted to their cytosolic fraction, as these proteins mainly displayed cytosolic pools.

1. Christoforou, A., Mulvey, C. M., Breckels, L. M., Geladaki, A., Hurrell, T., Hayward, P. C., Naake, T., Gatto, L., Viner, R., Martinez Arias, A., and Lilley, K. S. (2016) A draft map of the mouse pluripotent stem cell spatial proteome. *Nat. Commun.* 7, 8992
2. Itzhak, D. N., Davies, C., Tyanova, S., Mishra, A., Williamson, J., Antrobus, R., Cox, J., Weekes, M. P., and Borner, G. H. H. (2017) A Mass Spectrometry-Based Approach for Mapping Protein Subcellular Localization Reveals the Spatial Proteome of Mouse Primary Neurons. *Cell Rep.* 20, 2706-2718
3. Itzhak, D. N., Tyanova, S., Cox, J., and Borner, G. H. (2016) Global, quantitative and dynamic mapping of protein subcellular localization. *Elife* 5
