# Supplementary material for: Spatial Proteomics Reveals Differences in the Cellular Architecture of Antibody-Producing CHO and Plasma Cell–Derived Cells
Source: Mol Cell Proteomics. 2022 Aug 5;21(10):100278. doi: 10.1016/j.mcpro.2022.100278 (PMC9562429; doi:10.1016/j.mcpro.2022.100278)
Supplement: TableS1 [file mmc2.docx]

Table S1 Weighting factors for subcellular compartments

| Subcellular compartment | Fractions used as weighting factors |
| --- | --- |
| Cytosol | Cytosolic |
| Endoplasmic reticulum | Nuclear + organelle + cytosolic |
| Golgi apparatus | Nuclear + organelle |
| Lysosome | Nuclear + organelle + cytosolic |
| Mitochondrion | Nuclear + organelle |
| Nucleus | Nuclear + organelle + cytosolic |
| Peroxisome | Nuclear + organelle |
| Plasma membrane | Nuclear + organelle |
| Proteasome | Cytosolic |
| Ribosome | Nuclear + organelle + cytosolic |
